# Advances in protein-protein interaction prediction: a deep learning perspective

**DOI:** 10.3389/fbinf.2025.1710937

**Published:** 2026-01-07

**Authors:** Noor Alkhateeb, Mamoun Awad

**Affiliations:** College of IT, UAE University, Al-Ain, United Arab Emirates

**Keywords:** protein-protein interaction, deep learning, artificial neural networks, machine learning, bioinformatics

## Abstract

Protein–protein interactions (PPIs) are vital for regulating various cellular functions and understanding how diseases are developed. The traditional ways to identify the PPIs are costly and time-consuming. In recent years, the disruptive advances in deep learning (DL) have transformed computational PPI prediction by enabling automatic feature extraction from protein sequences and structures. This survey presents a comprehensive analysis of DL-based models developed for PPI prediction, including convolutional neural networks (CNNs), recurrent neural networks (RNNs), deep neural networks (DNNs), graph convolutional networks (GCNs), and ensemble architectures. The review compares their feature representations, learning strategies, and evaluation benchmarks, emphasizing their strengths and limitations in capturing complex dependencies and structural relationships. In addition, the paper elaborates on different benchmarks and biological databases that are commonly used in different experiments for performance comparison. Finally, we outline open challenges and future research directions to enhance model generalization, interpretability, and integration with biological knowledge for reliable PPI prediction.

## 1 Introduction

Protein-protein interactions play critical roles in many physiological activities, such as gene replication, transcription, translation, cell cycle regulation, signal transduction, immune response, etc. To understand and utilize these interactions, it is necessary to identify residues at the interaction interface (Zeng et al., 2016). Protein-protein interactions (PPIs) are pivotal in maintaining the integrity and functionality of cellular processes. These interactions mediate a variety of critical functions, including signal transduction, metabolic regulation, and the control of cell growth. As essential building blocks of cellular machinery, PPIs facilitate the coordination of numerous physiological and pathological processes. By studying PPIs, researchers can understand how proteins collaborate to modify enzyme kinetics, activate or suppress specific proteins, regulate molecular pathways, and even transport molecules across cellular compartments. The comprehensive mapping of PPIs, often referred to as the “interactome,” offers a profound insight into cellular functions and disease mechanisms. For example, the disruption of specific PPIs can lead to cellular dysfunction, making them an attractive target for therapeutic interventions, particularly in diseases like cancer, where altered signaling events are key drivers of tumorigenesis. Targeting PPIs provides a novel therapeutic approach by promoting or inhibiting these interactions to restore normal cellular behavior or inhibit disease progression. This strategy has shown promise in the development of new cancer therapies, aiming to interfere with the molecular interactions that enable cancer cells to thrive (Xia et al., 2016). In addition, PPI networks serve as a valuable resource for uncovering essential biological knowledge. By analyzing the interactions between proteins, researchers can gain a deeper understanding of cellular pathways, protein complexes, and their involvement in different diseases. Through this analysis, novel drug targets can be identified, which could lead to the development of more precise and effective treatments. Moreover, understanding the specific interactions between proteins in varying contexts, such as different cell types, developmental stages, or environmental conditions, is crucial to advance personalized medicine and improve therapeutic outcomes (Xia et al., 2016).

Studies have shown that the protein interaction interface is generally large; a typical interaction inter-face is about 1200–2000 Å^2^, but only a few (<5%) of the residues called hotspots contribute to most of the binding free energy and play an important role in the stability of protein binding (Moreira et al., 2007). The widely used databases of experimentally verified hotspots include the Alanine Scanning Energetics Database(ASEdb) (Thorn and Bogan, 2001), the Binding Interface Database(BID) (Fischer et al., 2003), the Protein-protein Interaction Thermodynamic (PINT) (Kumar and Gromiha, 2006), and the Structural Database of Kinetics and Energetics of Mutant Protein Interactions (SKEMPI) (Moal and Fern´andez-Recio, 2012). Experimental techniques for PPI identification, including yeast two-hybrid screening, co-immunoprecipitation, and tandem affinity purification, remain time-consuming, expensive, and prone to false positives or negatives. To overcome these challenges, computational prediction methods have emerged as efficient alternatives capable of large-scale analysis across proteomes. However, the complexity of protein structures, variability in data quality, and imbalance between positive and negative samples present major obstacles to achieving accurate and generalizable predictions. Computational methods thus aim to complement experimental studies by providing scalable, interpretable, and biologically relevant models that can prioritize candidate interactions for laboratory validation.

Computational PPI prediction can generally be divided into two core tasks. One is the prediction of putative interaction sites on the surface of an isolated protein, known to be involved in PPI sites prediction (PPISP), but where the structure of the partner or complex is unknown (Jones and Thornton, 1997). The second prediction problem is the prediction of pair-wise interactions to predict interfacial residues of a pair of proteins, which is related to the docking of two proteins. A large amount of PPI data for different species, generated through high-throughput experimental techniques, presents a significant challenge in data integration, noise reduction, and reliability assessment, making it a crucial area of research in computational biology. However, the absence of information about the partner proteins makes the latter also relatively more challenging (Ahmad and Mizuguchi, 2011).

Existing PPI prediction methods can be roughly divided into three types: knowledge-based methods, molecular simulation techniques, and machine learning methods (Deng et al., 2013). The knowledge-based empirical function evaluates the change in binding free energy by reducing the empirical model obtained using experiments. The introduced molecular dynamics model uses alanine to perform fixed-point scanning by the mutagenesis technique to detect the PPIs by examining the change of binding energy in the process of mutation to alanine. However, such an *in silico* technique is limited by factors such as the expense of the experimental equipment, the long computing time it takes, and the limited number of PPIs tested. Machine learning approaches provide a more convenient way for PPI prediction.

**FIGURE 1:**
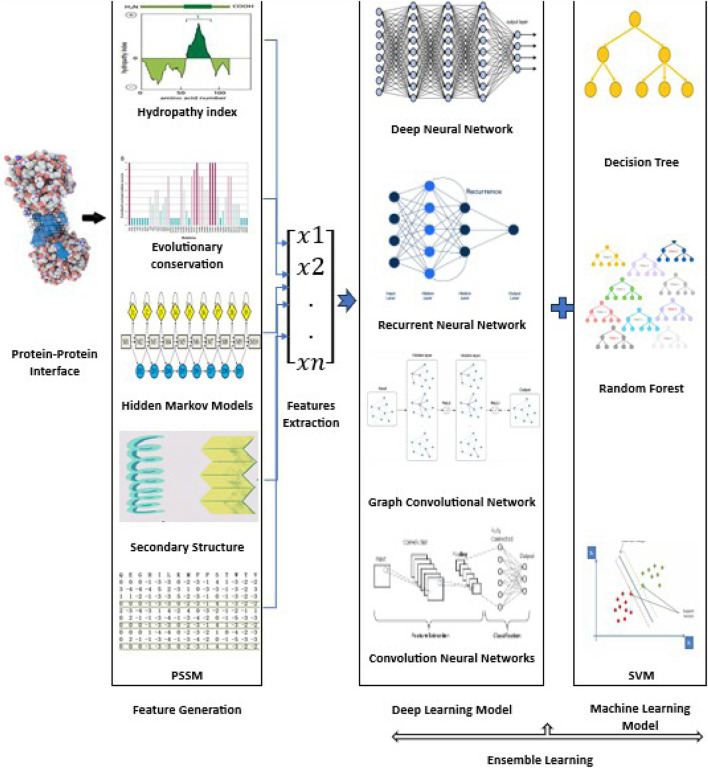
Overview of deep learning approaches to predict PPIs. For the binding of interface residues in PPIs, a large number and variety of features are extracted from diverse data sources. Then, feature extraction and feature selection approaches are used for dimensionality reduction. Finally, the deep learning-based prediction models are trained and applied to make predictions of PPIs. For some approaches, the machine learning model is attached to another deep learning model to complete the classification task.

The formation of a suitable feature set and the selection of favorable machine learning algorithms are two major stages in the development of prosperous predictions. The feature set can be constructed wisely in such a way that it could cover the maximum information or key features from the structure of the proteins. Among the structures, the primary structure, i.e., the sequences of the protein, is the most common to work on because of the availability of huge data (Wang L. et al., 2019). To extract protein interaction information, several feature extraction methods have been developed in the past to represent protein information in numerical forms (Jia et al., 2015; Cho et al., 2009). For PPI prediction, each feature extraction algorithm requires a favorable classifier to appropriately classify the interaction or no interaction according to the feature sets. The researchers applied various classification algorithms such as Random Forest (RF), Support Vector Machines (SVM), and their derivatives (Wei et al., 2016; Guyon et al., 2002), gradient boosting decision trees (Deng et al., 2013), and ensemble classifiers (Wang L. et al., 2019). Deep learning algorithms, which mimic the deep neural connections and learning processes of the human brain, have received considerable attention due to their successful applications in speech and image recognition (Guglani and Mishra, 2021), natural language understanding (Bacciu et al., 2021), and decision making (Silver et al., 2016). Compared to traditional machine learning methods, deep learning algorithms can handle large-scale raw and complex data and automatically learn useful and more abstract features (LeCun et al., 2015). In recent years, these algorithms have been applied in Bioinformatics to manage large high-dimensional data generated by high-throughput techniques (Zhang et al., 2016). Figure 1 shows typical applications of deep learning in PPI prediction. Usually, the input to the PPI predictor is a target interface residue that is encoded by a variety of sequence, structural, and energy features. Dimensionality reduction (feature selection or feature extraction) is then used to remove irrelevant/redundant information and obtain a set of principal variables. Finally, predictive models are built using efficient deep learning algorithms.

This paper provides an in-depth exploration of Deep Learning-based methods for predicting protein-protein interactions (PPIs), with a specific focus on sequence-based PPI prediction using deep learning (DL) models. In addition, we highlight key challenges and considerations when adopting these approaches, including feature generation, dimensionality reduction, and algorithm design. The paper classifies existing DL approaches based on factors such as extracted features, benchmark datasets, research contributions, model hyperparameters, and prediction performance, offering a comprehensive analysis of the strengths and weaknesses of widely used biological features and classical deep learning algorithms. The scope of this paper is primarily confined to the primary structure of proteins, i.e., the amino acid sequence, and its use in PPI prediction. For the first time, the significance of the protein’s primary structure and the approaches to representing protein sequences through deep learning are discussed in detail. The paper emphasizes the central importance of understanding protein sequences in the context of PPI prediction.

The paper is structured as follows: Section 1 introduces the concept of proteins and PPIs, explores the benefits of detecting PPIs, and provides an overview of recent advances in computational approaches within Bioinformatics. Section 2 discusses the different features that can be extracted from protein sequences, protein structure, and protein energy for PPI prediction. In Section 3, four prominent deep learning models are presented in addition to the ensemble learning models. Section 4 reviews research publications on sequence-based PPI prediction using DL, assessing their pros, cons, and performance outcomes. Section 5 offers a critical discussion on the effectiveness of deep learning in PPI prediction, and Section 6 concludes the paper and summarizes findings and potential future directions in this area of research.

## 2 Feature generation

Feature engineering is a crucial step in the development of effective PPI prediction approaches. Typically, raw data is transformed into features that have a significant impact on prediction performance. Often, a large number of features or attributes are collected from the protein sequence, structure, and energy data. Dimensionality reduction approaches are used to obtain the most effective features for future classification tasks.

### 2.1 PPI sequence-based features

#### 2.1.1 Position-specific scoring matrix (PSSM)

**FIGURE 2:**
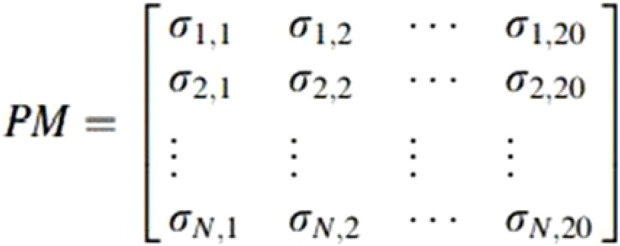
PSSM matrix.

**FIGURE 3:**
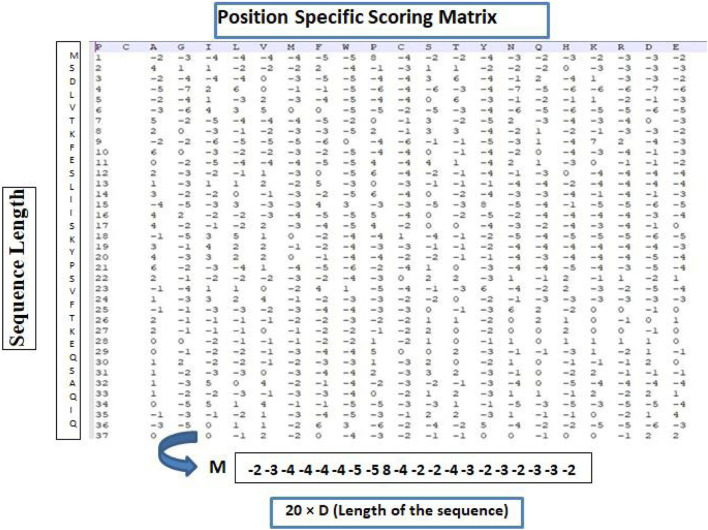
PSSM matrix of a protein sequence of length 37.

The position-specific scoring matrix (PSSM) is a kind of sequence matrix derived from multiple sequence alignment and captures the probability of amino acids or nucleotides occurring in each position. PSSM was introduced by Gribskov et al. (1987) to detect distantly related proteins. The rows in PSSM represent the position of residues in an alignment, and the columns specify the names of residues or amino acids. In protein sequences, PSSM has 20 columns representing the 20 amino acids. From a structural point of view, several amino acid residues could be mutated without altering the structure of the protein, making it possible that two proteins could share similar structures with different amino acid compositions. Figure 2 depicts the PSSM matrix structure, where σ(i,j) represents the probability that the *ith* residue was mutated into the *jth* amino acid during the evolutionary process. Position-Specific Iterated BLAST (PSI-BLAST) is a tool used to compute PSSM from the multiple sequence alignments of sequences scored above a certain threshold using protein–protein BLAST (Ahmad and Sarai, 2005). The PSSM is further updated by going through a set of iterations to search the NR database for new matches (Altschul et al., 1990). As such, each protein sequence is converted into a *N×*20 PSSM matrix where *N* is the length of the protein sequence. Figure 3 presents a snapshot of the PSSM matrix of a protein sequence of length 37.

#### 2.1.2 Evolutionary conservation

Evolutionary conservation indicates that similar genes or chromosome segments in different species reflect the common origin of a species, as well as important functional properties. Evolutionary conservation is computed by aligning the amino acid sequences of proteins with the same function and from different species. It can be calculated by computing the similarity between PSSM profiles of two proteins (Aybey and Gümüş, 2023), or by considering the mutual information by Detecting the co-evolving residues between two proteins (Hooft et al., 2008).

#### 2.1.3 Residue conservation

Residue conservation measures the frequency of specific amino acid residue in a protein is maintained across different species. This measure indicates its importance for both the protein’s structure and function. In isolated protein, sequence conservation is calculated per residue from the amino acid frequency distribution in the corresponding column of the multiple sequence alignment of homologous proteins. It can be computed by the STRUM method, which predicts the stability change caused by single-point mutations (Quan et al., 2016).

#### 2.1.4 Raw protein sequence

**FIGURE 4:**
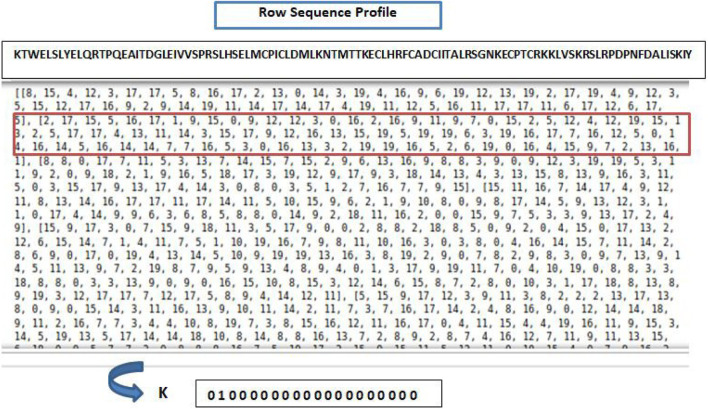
Raw protein sequence feature related to the first residue (K) of the mentioned protein sequence from the Dset 186 dataset.

Most proteins consist of 20 types of different amino acids. Thus, the 20xN one-hot encoding vectors are used to represent the positions of the amino acids in the proteins, where N is the length of the protein sequence. One-hot encoding (20-dimensional) is used so each residue is represented as a sparse binary vector where only one position is active, corresponding to the amino acid type. Figure 4 shows a snapshot of the raw protein sequence feature of the first residue of the mentioned protein sequence in the Dset 186 dataset (Murakami and Mizuguchi, 2010).

#### 2.1.5 Position information

This feature is used by some approaches such as D-PPIsite (Hu et al., 2023) and DELPHI (Li et al., 2021). The position information (PI) of each residue is modeled as one feature source to represent the feature of each residue. The PI of the i-th residue in the protein of N residues is calculated as i/N.

#### 2.1.6 High-scoring segment pair (HSP)

HSP is the local alignment that scores highest between two proteins. The similarities between two sub-sequences of the same length are measured by scoring matrices, such as PAM and BLOSUM. It can be calculated using SPRINT (Li and Ilie, 2017).

#### 2.1.7 The 3-mer amino acid embedding (ProtVec1D)

The concept of embedding is borrowed from natural language processing (NLP) where a word is represented by numeric vectors using techniques such as word2vec (Mikolov et al., 2013a). In Bioinformatics, protein vectors are based on ProtVec (Asgari and Mofrad, 2015), which also uses word2vec to construct a 100D for each amino acid 3-mer. ProtVec1D is a one-dimensional vector (1D) computed by summing the ProtVec components.

#### 2.1.8 Hidden markov models profiles (HMM)

The HMM profile can be produced by running HHblits v3.0.3 (Remmert et al., 2012) to align the query sequence against the UniClust30 database (Mirdita et al., 2017) with default parameters.

### 2.2 Structure-based features

Protein tertiary structure refers to the folding arrangement of amino acids in three dimensions, which can help to understand the function of proteins at the molecular level. Incorporating structural features can better apply the spatial structure features of proteins in PPIs prediction, and generally obtains better results than sequence-based features.

#### 2.2.1 Secondary structure

**FIGURE 5:**
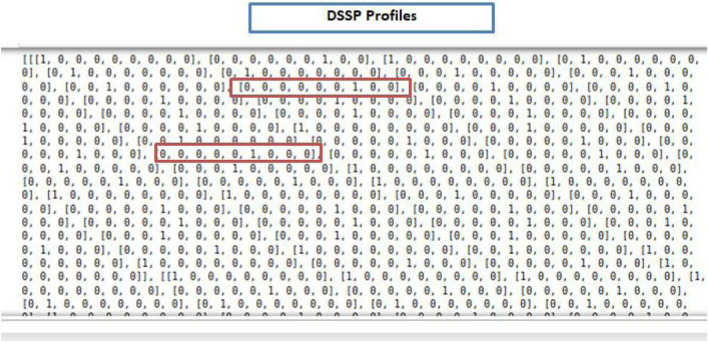
Protein secondary structure for each residue in a protein sequence extracted by DSSP.

The protein secondary structure depicts the regular folding or local spatial structure of regions within one polypeptide chain. It is very common to encode structural information of amino acids in PPIs prediction. Secondary structure is typically generated by tools such as DSSP (Zeng et al., 2020). In DSSP, there are eight categories of secondary structures: 310-helix (G), a-helix (H), p-helix (I), b-strand (E), b-bridge (B), b-turn (T), bend (S) and loop or irregular (L). Considering that some amino acids do not have their secondary structure stated in the DSSP file, thus 9D one-hot encoding vector is used to encode the secondary structure. The first eight dimensions, in the 9D one-hot vector, represent the state of each amino acid, and the last dimension represents the absence of such information. Each protein sequence can be converted into an N × 9 DSSP matrix, where N is the length of the protein sequence. Figure 5 shows a snapshot of the secondary structure features of the sample annotated protein sequences.

#### 2.2.2 Relative solvent accessibility metrics (RSA)

This feature is also calculated by the DSSP library. RSA reflects the fraction of a residue that is exposed to a potential solvent. This is computed by sliding a spherical probe of the radius of 1.4Å [approximating the radius of a water molecule (Eisenhaber et al., 1995)] over the Van Der Waals surface of the protein near the residue of interest. The Area generated by the center of the probe, as it is in contact with the residue, is taken to be the accessible surface area. This is divided by the maximum possible accessible surface area to achieve a relative measure. Concretely, it can be predicted by subsequence artificial neural network (SANN) (Joo et al., 2012), for each query sequence, the RSA profile (N x 3 matrix, where N is the length of the query sequence) includes the probabilities of three solvent accessibility classes (i.e., buried (B), intermediate (I), and exposed (E)).

#### 2.2.3 PKx

PKx is a property of amino acids that measures the dissociation constant (K_*D*_), which is the propensity of an amino acid to separate (dissociate) into smaller components. It is calculated by applying the negative of the logarithm of the dissociation constant for any other group in the molecule (Zhang B. et al., 2019; Li et al., 2021).

#### 2.2.4 3D-1D scores

The side-chain environment was first proposed by Eisenhaber et al. (1995) and used in the 3D-profile structural prediction method. 3D-1D scores are a feature that quantifies the mismatch between the residue local environment in 3D structure and its sequence context (1D). For each residue, a structural environment descriptor is computed (e.g., RSA, contact density, secondary structure) and compared with the corresponding position in the 1D sequence (amino acid properties). The score is computed as a normalized difference or similarity between these representations (Matsuo et al., 1995). Authors in Fan et al. (2016) utilized it for the prediction of protein solvent accessibility.

### 2.3 Hybrid features

This section includes features derived from amino acid sequences, but they are inherently linked to residue-level structure and folding. We classified these features hybrid, representing physicochemical tendencies that bridge sequence and structure spaces.

#### 2.3.1 Physical properties

Some approaches extract the physical properties to represent the protein sequence (Meiler et al., 2001). The seven-dimensional physical properties are as follows: a steric parameter (graph shape index), polarizability, volume (normalized van der Waals volume), hydrophobicity, isoelectric point, helix probability, and sheet probability.

#### 2.3.2 Hydropathy index

A number that represents the Hydrophobicity scale. It is typically composed of experimentally determined transfer-free energies for each amino acid, as well as it is essential to understand the energetics of protein-bilayer interactions (Wimley and White, 1996; Kyte and Doolittle, 1982).

#### 2.3.3 Physicochemical characteristics

Protein physicochemical characteristics include the number of atoms, electrostatic charges, potential hydrogen bonds, hydrophobicity, hydrophilicity, side-chain volume, polarity, polarizability, solvent-accessible surface area (SASA), and side-chain net charge index (NCI) (Zhang B. et al., 2019).

### 2.4 Energy-based features

#### 2.4.1 Relative amino acid propensity (RAA)

The amino acid propensity for binding is quantified as the relative difference in abundance of a given amino acid type between binding residues and the corresponding non-binding residues located on the protein surface (Li et al., 2021; Aybey and Gümüş, 2023).

#### 2.4.2 Van Der Waals energy

The Van Der Waals energy reflects the weak, non-covalent interactions between non-bonded atoms. It is important in modeling the steric (spatial) compatibility between protein surfaces (Meiler et al., 2001).

### 2.5 Feature selection

Feature selection can provide a deeper insight into the underlying means that generate the data, avoid overfitting, and improve the prediction performance. Typical feature selection algorithms include Fisher’s Score (F-score) (Chen and Lin, 2006), random forest (Wei et al., 2016), and support vector machines–recursive feature elimination (SVM-RFE) (Guyon et al., 2002). Several feature selection approaches have been used for PPI prediction. APIS (Xia et al., 2010) used the F-score, while the authors in (Cho et al., 2009) used a decision tree to select relevant and useful features. Qiao et al. (2018) developed a hybrid feature selection strategy that combines the F-score, mRMR (minimum redundancy maximum relevance), and the decision tree to select the features.

### 2.6 Feature extraction

Feature extraction is the process of converting raw data into numeric data or features. In many machine learning applications, feature extraction techniques are used to select the most relevant features by reducing the dimensionality of a dataset. Principal component analysis (PCA) (Jia et al., 2018) and linear discriminant analysis (LDA) (Mika et al., 1999) are two commonly used feature extraction techniques. PCA works by establishing an orthogonal transformation of the data to convert a set of possible correlated variables into a set of linearly uncorrelated ones, the so-called principal components. LDA can help improve the accuracy of predictions by reducing the dimensionality of high-dimensional data while retaining discriminative information.

## 3 Deep learning models

The selection of an appropriate DL technique plays an important role in improving the performance of PPI prediction. This review mainly considers four DL architectures: Deep Neural Networks (DNN) (Zhang et al., 2016), Convolutional Neural Networks (CNN) (Zeng et al., 2020), Recurrent Neural Network (RNN), and Graph Convolutional Network (GCN) (Yuan et al., 2021). In addition, we consider ensemble learning (EL) techniques (Wang X. et al., 2019), which combine several learning models in one. These architectures have been widely used in PPI prediction in recent years. This section provides the reader with brief overview about these architectures.

### 3.1 Deep neural networks (DNN)

**FIGURE 6:**
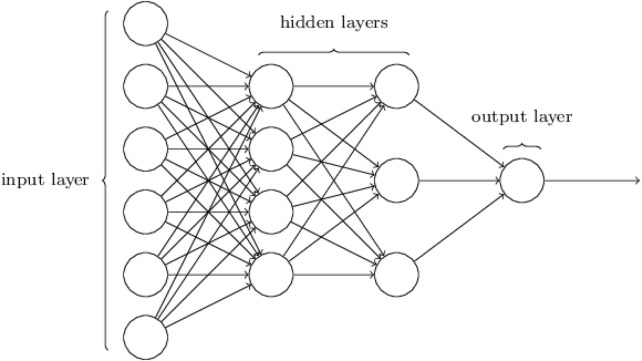
Basic structure of DNNs with one input layer, two hidden layers, and one output layer. At each layer, the weighted sum and non-linear function of its inputs are computed to obtain an abstract representation.

DNNs typically consist of more than one hidden layer, organized in deeply nested network architectures. Furthermore, they usually contain advanced neurons in contrast to simple Artificial Neural Networks (ANNs). That is, they may use advanced operations (e.g., convolutions) or multiple activations in one neuron rather than using a simple activation function (Li et al., 2022). The output of a specific layer can be calculated as in Equation 1:

$$P_{x}^{\left(n+1\right)}=\mu \left(W_{x}^{n+1}{\;P}^{n}+Z_{x}^{n+1}\right)$$ (1)

where *µ* presents the activation function, W is the weight matrix, *P* ^*n*^ is the inputted data for the *n* ^*th*^ layer and Z is the bias term (Guglani and Mishra, 2021). These characteristics allow DNNs to be fed with raw input data and automatically discover a representation that is needed for the corresponding learning task. Adding more hidden layers to the network to learn from raw data is the core capability of DNN to learn complex tasks; hence its name DL, see Figure 6.

### 3.2 Convolutional neural network (CNN)

**FIGURE 7:**
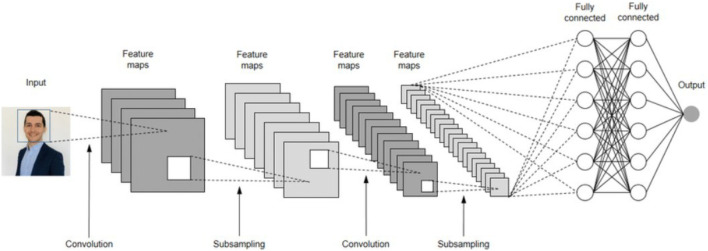
The structure of CNN.

A CNN is a type of DL algorithm that processes input in the form of images, assigning learnable weights and biases to various features. This enables CNNs to distinguish between different images with minimal pre-processing compared to other classification algorithms (Wang L. et al., 2019). Structurally, a CNN is a feed-forward neural network where neurons respond to neighboring units within a defined coverage area, and it excels in data feature extraction (Albawi et al., 2017). The output is calculated using forward propagation, and weights and biases are adjusted through backpropagation. Figure 7 illustrates the structure of a CNN, which consists of the input layer, convolutional layer, subsampling layer, fully connected layer, and output layer. The feature map *M*_1_ at the *l* ^*th*^ layer is computed as in Equation 2 (Albawi et al., 2017):

$$M_{l}=f\left(M_{l-1}⋆W_{l}+b_{l}\right)$$ (2)

where *W*_*l*_ is the weight matrix of the convolution kernel of *l* ^*th*^ layer, *b*_*i*_ is the offset vector, *f* represents the activation function, and ⋆ denotes the convolution operation. The subsampling layer, which is usually located behind the convolutional layer and the feature map, is sampled according to the following rules. Suppose *M*_*l*_ is a subsampling layer, which is formulated as in Equation 3:

$$M_{l}=subsampling\;\left(M_{l}-1\right)$$ (3)

The fully connected layer is responsible for the classification of the extracted features via several convolution and subsampling operations. The fundamental mathematical notion of CNN is to map the input matrix *M*_*o*_ to a new feature representation *R* through multi-layer data transformation, see Equation 4.

$$R\left(l\right)=Map\;\left(C=c_{1}\;|M_{O}\;;\;\left(w,b\right)\right)$$ (4)

where *c*_*l*_ represents the *l* ^*th*^ label class, Mo denotes the input matrix, and R denotes the feature expression. The goal of CNN training is to minimize the network loss function *R(w, b)*. At the same time, to ease the overfitting problem, The final loss function *Z(w, b)* is usually controlled by a norm, and the intensity of the overfitting is controlled by the parameter ϵ, see Equation 5.

$$Z\left(w,b\right)=R\left(w,b\right)+\frac{\varepsilon }{2}w^{T}w$$ (5)

While CNNs are traditionally used for images, in PPI prediction, they handle structured numerical data derived from protein sequences, structures, or energy values. CNNs are particularly effective at capturing local patterns, making them suitable for identifying interaction motifs or residues crucial for binding.

### 3.3 Recurrent neural network (RNN)

**FIGURE 8:**
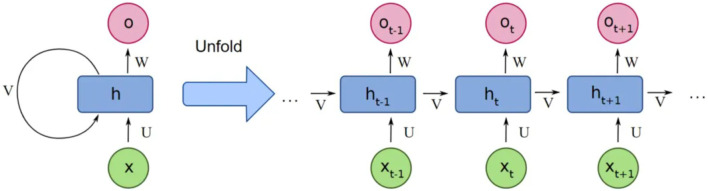
Basic structure of RNNs with an input unit x, a hidden unit h and an output unit O. The recurrent computation can be expressed more explicitly if the RNNs are unrolled in time. The index of each symbol represents the time step. In this way, h_*t*_ receives input from x_*t*_ and h_*t−*1_ and then propagates the computed results to O_*t*_ and h_*t*+1_.

The structure of RNNs has a recurring link in each hidden layer, which is responsible for operating sequential information by some recurrent computation as shown in Figure 8. The previous output (state vector) is kept in hidden units, and for the current state, the output is calculated using the previous state vector and the considered input (Li et al., 2021). The evolution of RNN over time is expressed as in Equations 6, 7 below (Richoux et al., 2019):

$$O_{t}=\delta \left(h_{t};\;\theta \right)$$ (6)

$$h_{t}=g\left(h_{t-1},x_{t};\;\theta \right);$$ (7)

here, *θ* includes weights and biases for the network, the first equation expresses the dependency of the output *O*_*t*_ at time t only with the hidden layer *h*_*t*_ using some computation function *δ* and the second equation shows the dependency of the hidden layer *h*_*t*_ at time *t* with that of *h*_*t−*1_ at time *t* *−* 1 and the input *x*_*t*_ at time *t*. RNNs can be used effectively in PPI prediction due to their ability to process sequential data. Since protein sequences are essentially linear chains of amino acids, RNNs are well-suited for capturing the sequential dependencies and long-range interactions between residues (Richoux et al., 2019).

### 3.4 Graph convolutional network (GCN)

Graph Neural Networks (GNNs) are structured graphs built from generalizing neural networks to work on arbitrarily structured graphs. GCN was developed to solve many bioinformatics problems. Defining parameterized filters that are used in a multi-layer GNN leads to GCNs. Currently, most GNN models have a relatively universal architecture in common. It is convolutional because filter parameters are typically shared over all locations in the graph. In Protein-Protein Interaction (PPI) prediction, proteins can be modeled as nodes in a graph, where edges represent potential interactions between them. GCNs are especially well-suited for this task because they operate directly on graph-structured data, capturing the relational dependencies between proteins more effectively than traditional models. The layer-wise propagation rule for a GCN is given in Equation 8.

$$H^{\left(l+1\right)}=\sigma \left({\tilde{D}}^{-\frac{1}{2}}\tilde{A}{\tilde{D}}^{-\frac{1}{2}}{\;H}^{l}{\;W}^{l}\right)$$ (8)

**FIGURE 9:**
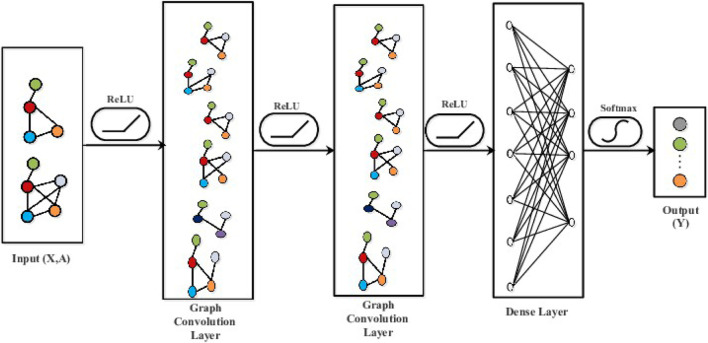
The structure of GCN.

Where *H* ^*l*^ is the matrix of node representations at layer *l, H* ^0^ is the input feature matrix, *Ã = A + I* is the adjacency matrix *A* of the graph with added self-loops (identity matrix *I*), *D* ^˜^ is the degree matrix of *Ã*, *W* ^*l*^ is the weight matrix of the lth layer, and σ is the activation function. Figure 9 illustrates the GCN structure.

### 3.5 Ensemble learning (EL)

Ensemble learning is a powerful machine learning technique that involves the combination of multiple models to improve overall performance, particularly in tasks such as classification, regression, and prediction. Rather than relying on a single model, ensemble learning leverages the strengths of various models to create a more robust and accurate final prediction. The idea is based on the principle that a group of weak learners (models that perform slightly better than random guessing) can be combined to form a strong learner. By combining the three deep learning models (DNN, CNN, and GCN) with traditional machine learning algorithms, researchers aim to build more comprehensive models that can better predict PPIs by taking advantage of both high-level feature learning and well-established traditional machine learning techniques.

**FIGURE 10:**
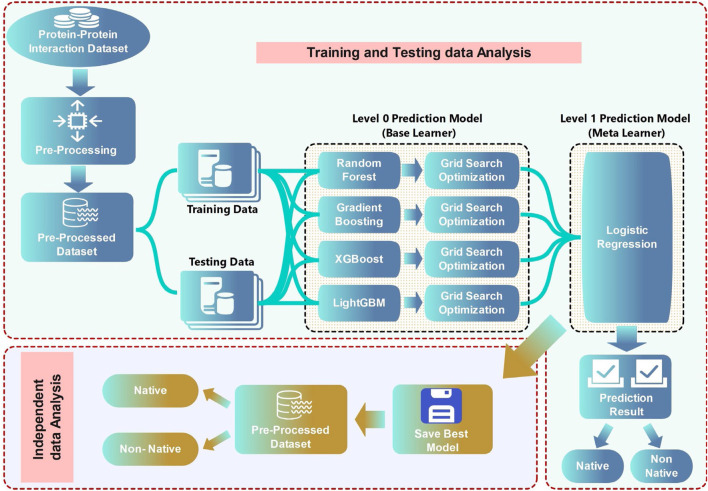
Example of Ensemble classifier (Pratiwi et al., 2024).

Figure 10 illustrates a schematic representation of a two-tier machine learning framework to classify protein-protein interactions. The training data are used to build and optimize several base learners, including random forest, gradient boosting, XGBoost, and LightGBM, through grid search optimization. A meta-learner, logistic regression, takes the prediction of these models to generate the final classification results (Pratiwi et al., 2024).

## 4 PPI prediction approaches using deep learning models

**FIGURE 11:**
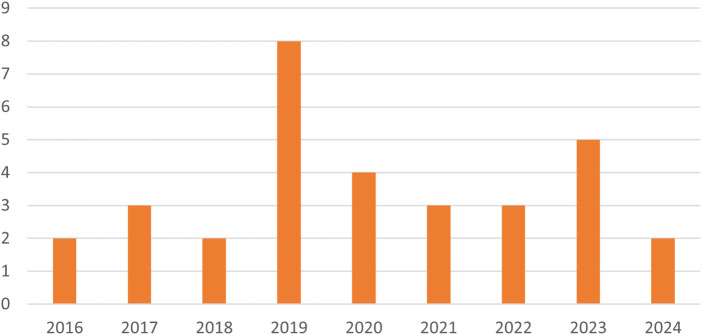
Yearly publication analysis of PPI prediction using Deep Learning.

This section summarizes existing deep learning-based approaches for PPI identification. Firstly, we will explore these approaches from the perspective of protein shape, focusing on two key approaches, namely, Approach A: site prediction of an isolated protein and Approach B: prediction of PPI for a pair of proteins. To date and to the best of our knowledge, there are around 32 research papers that have been published for PPI prediction using DL, see publication analysis in Figure 11. In this section, we will elaborate on the studies performed on PPI prediction tasks using DL. The summary of these studies can be found in Table 1. We examined various feature representations, including sequence-based, structure-based, and physicochemical properties, to enhance the understanding and prediction of PPI dynamics. The research studies in Table 1 are classified based on: year of publication, research contribution, approach type, dataset type, input features, and hyperparameters of the network. The term “Approach” is written after each section to indicate the category of the approach in the table. All important abbreviated terms of the table are provided in expanded form in the corresponding text, whereas the basic abbreviations are provided after the abstract. The detailed description of this section is broadly divided based on both approaches. For better readability and to minimize confusion about abbreviations, Table 2 summarizes the datasets that were considered for Approach A, and Table 3 lists the datasets for Approach B, as well as the cited papers in subsequent sections.

**TABLE 1:** Deep learning methods for protein-protein interaction sites prediction.

Year [References]	Approach DL model	Research contribution	Datasets	Input features	Hyper parameters	Highest accuracy	Limitation
Approach A							
Wei et al. (2016)	SSWRF EL (RF + SVM)	Dealing with class imbalance	A, B, and C	PSSM, ACH, and ACRSA	N/A	67.90 (A)	Fails to generalize across datasets; over-sampling may intro-duce noise
Jia et al. (2016)	iPPBS-Opt EL (KNN + IHTS)	Optimizing imbalanced training datasets	surface-residue and all-residue	hydrophilicity, side-chain volume, polarity, polarizability, SASA, and NCI	N/A	85.45 (All residue)	Parameter tuning, Model complexity due to multiple pre-pre-processing steps
Zhang B et al. (2019)	DLPred DNN	Improving the imbalanced prediction of PPI	A, B, C, and D	PSSM, physical properties, hydropathy index, physicochemical characteristics, PKx, 3D-1D scores, conservation score, raw protein sequence	Output dimensionality of each layer = 400, sixty-four hidden nodes, AF: softmax, dropout (p = 0.5)	81.81 (D)	Capturing long range dependencies, overfitting on small datasets
Wang X et al. (2019)	ELSMURF EL	Dealing with the imbalance Problem	A, B, and C	PSSM-SPF and RER	Sliding Window = 11, (3,5,7) RFs	79.10	
Zeng et al. (2020)	DeepPPISP CNN	combining local contextual and global sequence features	A, B, and C	PSSM, secondary structure, raw protein sequence, RSA, polarity and protein sequence length	cross-entropy loss, Adam Optimizer, sliding window = 7, length of protein sequence = 500, batch size = 64; learning rate = 0.001; dropout rate = 0.2	81.81 (D)	
Xie et al. (2020)	CNN	Using residue binding propensity to improve the positive samples	I and J	AAC, PSSM, PSFM, Amino Acid Physicochemical Properties, ASA, RASA, geometric properties (CX, DPX), hydrophobicity	Three convolutional, pooling layers, one fully-connected layer, AF: Softplus and Softmax, Adam Optimizer	AUC = 91.20	
Yang et al. (2021)	PhosIDN DNN	Merging the local patterns and the long-range dependencies from protein sequences integrating DNN in the prediction of phosphorylation sites from protein sequences	G PPIs from the STRING database	N/A	Dropout layers are used in convolutional and fully connected layers, Adam optimizer	65.00	
Li et al. (2021)	DELPHI EL (CNN + RNN)	Features used for the first time	A, B, C, E, and F	HSP, position information, ProtVec1D, PSSM, evolutionary conservation, RSA, RAA, Putative protein-binding disorder, Hydropathy index, Physicochemical characteristics, Physical properties, and PKx	Convolution and bidirectional gated recurrent units (GRU) layers, sliding window = 31, lengths of protein sequence from 100 to several thousand, AF: Sigmoid	84.80 (F)	Limited generalization on unseen proteins with low homology
Mahbub and Bayzid (2022)	EGRET Graph Attention Netwerk + Prot-Bert PLM	First in using transfer learning in PPI site prediction, Using ProtBert PLM	A, B, and C	PSSM, Secondary Structure, evolutionary conservation, and RSA	AF:sigmoid, softmax normalization applied to generate a normalized attention score	71.5	Depends on pretrained embeddings, limited interpretability
Hu et al. (2023)	D-PPIsite DNN	Integrating multiple deep learning models to predict PPI Reducing the risk of a single prediction model getting stuck in local optima	A, B, C, E and F	PSSM, RSA, position information, and physical properties	One convolution layer, one squeeze and excitation layer, one batch normalization layer, one ReLU layer, five basic residual blocks layers (three convolution layers, two ReLU layers, and three BN layers), one average pool layer, one flatten layer and one fully connected layer with sigmoid	87.1 (F)	Limited scalability to large datasets
Kang et al. (2023)	HNPPISP CNN	Integrating CNN, MLPMixer, and LSTM models Combining local and global features	A, B, C, and H	Sub-sequences, sub-PSSM, and sub secondary structures	Sliding window = 7; per-patch FC = 512; LF: cross-entropy loss, optimization: Adam; learning rate: 0.1, 0.01, 0.001; batch size: 64, 128, 256; dropout: 0.2, 0.5, 0.7	66.70	Computationally expensive, Prone to feature redundancy, limited interpretability
Aybey and Gümüş (2023)	SENSDeep EL (CNN + RNN + GRU)	Improving the performance, better execution	A, B, C, E, and F	PSSM, HSP, ProtVec1D, Position Information, ECO, RSA, RAA, Putative Protein-Binding Disorder, Hydropathy Index, Physicochemical Characteristics, Physical Properties, PKx, Protein Sequence Information, Secondary Structure Information	N/A	83.40 (F)	Computationally expensive, limited interpretability of feature importance
Alkhateeb and Awad (2024)	DeepGCN GCN	Improving the accuracy, using new features	A, B, and C	PSSM, Protein Sequence Information, Secondary Structure, RSA, XYZ positions	5 hidden layers, 2 output dimensions, batch size = 32, learning rate = 0.0001, dropout fraction = 0.58, AF: ReLU	79.00	
Feng et al. (2024)	DGCPPISP GCN	Applying transfer learning to encoding the proteinsequence	SPRINTStr (Taherzadeh et al., 2018), A, B, C, E, and K	PSSM, HSP, One-hot Encoding, Cooccurrence Similarity Encoding, Similarity Encoding of Electrostaticity and Hydrophobicity, Position Encoding, and ESM2 Encoding	Conv encoder length = 3, padding parameter = 1, AF: LeakyReLU, negative slope parameter = 0.2, Batch Normalization, dropout = 0.5, neighborhood size = 10, multilayer perception = 64, convolution kernel size = 1, optimizer = Adam, learning rate = 0.01, batch size = 32, the number of hidden layers = 3	77.8 K	Computationally expensive
Approach B							
Sun et al. (2017)	DNN	First to apply a DL to PPI prediction. Applying stack auto encoder	A, B, C, D, E, F, G, H, I, J, and K	Physical-chemical properties	N/A	99.21 C	
Du et al. (2017)	DeepPPI DNN	Using two separate networks as input so the neural networks can better learn the characteristics of each protein	B, E, H, C, I, K, Q, R, and P	AAC, DPC, and APAAC	Learning rate = 1, Batch size = 16, Momentum rate = 0.8, AF: ReLU, and dropout = 0.1	98.14 Q	Limited transfer learning capacity, structural generalization
Wang et al. (2017)	EL (RF + DCT)	Applying DCT algorithm in PPI	E, P, M, I, K, Q, and R	PSSM, DCT	N/A	98.87 Q	
Li et al. (2018)	DNN-PPI DNN	Generalization tool for identifying protein interactions	A, C, F, G, I, J, K, and R	Semantic associations between amino acids position related sequence segments, LSTM dependencies	3 layered CNNs, AF: ReLU, and the length of the max-pooling = 2	98.66 K	
Hashemifar et al. (2018)	DPPI CNN	The ability to predict homodimeric Interactions	S, M, O, Q, R, V, J, K, X, Y, Z, and I	N/A	five convolutional modules, size of pooling window = 4, learning rate = 0.01, AF: ReLU	96.68 O	
Richoux et al. (2019)	DNN, RNN	Reliable dataset	N	AAC	AF: ReLU and sigmoid, LSTM layer = units, Adam optimizer, learning rate = 0.001	92.89 C	
Wang L et al. (2019)	CNNFSRF EL (CNN, RF)	Combining CNN with Feature-Selective Rotation Forest (FSRF) classifier	M and P	PSSM	N/A	98.65 Q	
Chen et al. (2019)	PIPR RCNN	Employing a Siamese architecture to capture the mutual influence of a protein sequence pair	E, N, K, I, J, Q, and W	N/A	Number of occurrences for the RCNN units = 5, the hidden state sizes are: 10, 25, 50, 75, cross-entropy loss, learning rate = 0.001, batch size = 256	97.90	
Wang Y et al. (2019)	Bio2Vec CNN	Considering the context information and implicit semantic information of the bio sequence	C, O, and P	N/A	N/A	97.31 C	
Zhang L et al. (2019)	EnsDNN EL (multi DNNs)	Leveraging multiple DNNs with different Configurations	O, K, I, Q, R, and P	AC, MCD, and LD	27 DNNs, AV: Relu, Dropout: 0.7, 0.5, Adam optimizer, Learning rate = 0.001	95.10 I	Risk of overfitting
Jia et al. (2015)	iPPIPseAAC EL (RF + CGR)	Incorporating the information of CGR into the PseAAC	P and O	PseAAC	N/A	92.95 P	
Chen et al. (2020)	StackPPI EL (RF + ET)	The ability to infer biologically significant PPI networks Using XGBoost to eliminate the noise and reduce the dimensionality	E, P, Q, R, K, I, Wnt-related pathway, and disease specific datasets	AAC, Morean-Broto, Moran, and Geary autocorrelation descriptor, Sequence information, AAC-PSSM, Bi-PSSM	N/A	98.71 I	Training complexity
Yang et al. (2020)	S-VGAE GCN	Involving Structural information of PPI networks in a graph with the sequence information to be informative in PPI prediction	C, E, J, I, K	AC	2 hidden layers, Adam Optimizer, learning rate = 0.005, AF: ReLU	99.80 J	
Czibula et al. (2021)	AutoPPI EL (multiple DNNs)	Applying Siamese architecture in both the encoder and the decoder	C, and Multi-species dataset	AC, and CT	DNN layers, AF: SELU, learning rate = 0.0005, batch size = 64	98.29 Multispecies dataset	
Le and Kha (2022)	DNN	Utilizing the FASTA (Pearson format) of amino acids	L and M	AAC, sequence-order information, and Topological Information	Three different dense layers were used here, including 100, 100, and 200 filters for each layer, respectively, AF: ReLU and sigmoid	94.10 C	
Li et al. (2022)	SDNNPPI DNN	Self-attention approach	O, T, S, Ab	AAC, Conjoint triad, and Auto covariance	AF: Sigmoid, six fully connected Layers	98.94 S	
Baranwal et al. (2022)	Struct2Graph GAN	Using a 3D-structurebased GAN in PPI prediction for the first time	O, Q, I, K,	N/A	Optimizer: Adam, learning rate = 0.001, rate-decay = 0.5 per 10 epochs, total epochs: 50, number of GCN layers = 2, GCN embedding dimension = 20, lLF: binary cross-entropy	98.96	High pre-processing cost
Tran et al. (2023)	DeepCFPPI DNN	Combining the learned features and handcrafted features for the first time	C, M, K, I, Q, P, R, One-core network (CD9), Wnt-related pathway crossover network (Wnt), and Cancer specific network	AAC, PseAAC, APAAC, QSO, DPC, and NLP learned features	LF: entropy, Adam optimization, learning rate = 0.001, AF: ReLU	100 I, K, P, Q, R, Wnt, cancer, CD9	Extensive feature engineering; computationally expensive
Zhang et al. (2023)	DeepSG2PPI CNN	Combining two CNNs Global features between the protein and GO function annotation is used to represent the biological features	GO, UniPort, and STRING databases	Sequence information, sequence local context information, and global statistics information	Dropout = 0.45, AF: LeakyReLU, Adam optimization, learning rate = 0.001	98.37	

**TABLE 2:** Short names given for datasets considered by cited papers in Approach A.

S. No	Dataset	Short name	Binding sites	Non-binding sites	References
1	Dset_186	A	5551	30,665	Murakami and Mizuguchi (2010)
2	Dset_72	B	3799	14,176	Murakami and Mizuguchi (2010)
3	Dset_164	C	6111	27,567	Singh et al. (2014)
4	heteromeric Dset_48	D	-	-	Zhang B et al. (2019)
5	Dset_448	E	15,810	100,690	Zhang and Kurgan (2019)
6	Dset_355	F	11,467	84,473	Li et al. (2021)
7	Kinase	G	-	-	Luo et al. (2019)
8	Dset_338	H	-	-	Kang et al. (2023)
9	protein-protein docking (DBD) v5.0	I	-	-	Vreven et al. (2015)
10	protein-protein docking (PBD) v4.0	J	-	-	Xie et al. (2020)
11	Dset_331	K	11,255	72,420	Kang et al. (2023)

**TABLE 3:** Short names given for datasets considered by cited papers in Approach B.

S. No	Dataset	Short name	References
1	Pan	A	Pan et al. (2010)
2	Swiss-Prot	B	Sun et al. (2017)
3	2010 HPRD	C	Huang et al. (2015)
4	2010 HPRD NR	D	Huang et al. (2015)
5	DIP	E	Li et al. (2018)
6	HIPPIE	F	Sun et al. (2017)
7	InWeb in BioMap	G	Sun et al. (2017)
8	2005-Martin	H	Martin et al. (2005)
9	*E. coli*	I	Zhou et al. (2011)
10	D.melanogaster	J	Das and Yu (2012)
11	*C. elegans*	K	Zhou et al. (2011)
12	HURI	L	Luck et al. (2020)
13	Yeast	M	Wang L et al. (2019)
14	Uniprot	N	Richoux et al. (2019)
15	*S. cerevisiae*	O	You et al. (2014)
16	*H. pylori*	P	Zhou et al. (2011)
17	*Homo sapiens*	Q	Zhou et al. (2011)
18	*Mus musculus*	R	Zhou et al. (2011)
19	human	S	You et al. (2013)
20	Human-Y.pestis	T	Kösesoy et al. (2019)
21	S.pombe	V	Das and Yu (2012)
22	SKEMPI	W	Moal and Fern´andez-Recio (2012)
23	A.thaliana	X	Das and Yu (2012)
24	B.subtilis	Y	Das and Yu (2012)
25	B.taurus	Z	Das and Yu (2012)
26	R.norvegicus	Aa	Das and Yu (2012)
27	Human-B.Anthracis	Ab	Das and Yu (2012)

### 4.1 Approach A: PPI prediction in isolated protein sequence

The PPI prediction in isolated protein sequences is crucial to identify potential interaction sites without requiring structural or pairing information. This method enables early-stage interaction analysis, which makes it valuable for large-scale screening and understanding intrinsic protein properties. Several studies have explored sequence-based PPI prediction, emphasizing its effectiveness in functional annotation and large-scale analysis. The authors in (Xie et al., 2020) leveraged the residue binding propensity to refine positive samples and introduced a context-based binding (CBB) approach for PPI site prediction, achieving remarkable results. In addition, it yielded much better results on samples with a high binding propensity than on randomly selected samples. Their findings indicated the presence of false-positive PPI sites due to distance-based residue definitions.

To enhance the prediction of the PPI site, some approaches proposed the combination of local and global features. Zeng et al. (2020) proposed DeepPPISP, a CNN-based framework that integrates local contextual and global sequence features. For local contextual features, a sliding window-based method is applied to extract features of the neighbors of an amino acid. By integrating local contextual and global sequence features, DeepPPISP achieved a good performance. The DeepPPISP was the first approach that combined the local contextual and global sequence features and showed that global sequence features played important roles in PPI site prediction.

In another advancement, the authors in Yang et al. (2021) developed PhosIDN, a DNN model for phosphorylation site prediction, integrating local patterns and long-range dependencies from protein sequences. PhosIDN consists of three closely connected sub-networks, including a sequence feature encoding sub-network (SFENet), a PPI feature encoding sub-network (IFENet), and a heterogeneous feature combination sub-network (HFC-Net). Comprehensive experiments were conducted to investigate the performance of this approach, and the evaluation results demonstrated that it improved the prediction performance of phosphorylation sites. Fur-thermore, by extracting features for the first time, Li et al. (2021) introduced an ensemble learning method for PPI prediction (DELPHI). It combined a CNN and an RNN structure with a fine-tuning technique. They used 12 feature groups to represent protein sequences, including 3 novel features (used for the first time in PPI prediction), HSP, position information, and a reduced 3-mer amino acid embedding (ProtVec1D). DEL-PHI outperformed the competitors in all metrics on all datasets, although it shared the least similarities to the testing datasets. In addition, DELPHI’s predicted PBR sites closely match known data from Pfam (El-Gebali et al., 2019). To address the problem of an imbalanced dataset, Zhang B. et al. (2019) developed a DL architecture (DLPred) based on an SLSTM network. The Experimental results showed that the model has improved F-measures, predictive accuracies, and AUC values. Compared with other predictors, DLPred is simple but more generalizable and one of the most popular solutions to improve the performance of imbalance classification. Followed by that in the same year, Wang X. et al. (2019) tackled the imbalance problem using EL-SMURF, an ensemble learning approach combining the synthetic minority oversampling technique (SMOTE) and Random Forest to oversample interfacial residues. SMOTE and the RF methods have been integrated to oversample interfacial residues in the feature space by generating new data from two types of sample data. They were the first who apply the fusion of sequence profile features in PSSM (PSSM-SPF) and residue evolution rate (RER) for feature extraction of neighboring residues with a sliding window. SMOTE was then applied to oversample interface residues in the feature space to deal with the imbalance problem. Then, they opti-mized the parameters of RFs and selected a different number of decision trees for different classifications by the leave-one-out cross-validation. Finally, the ensemble learning model was obtained by integrating the above-optimized RF classifier. Similarly, to solve the imbalance problem (Wei et al., 2016), proposed an ensemble model of SVM and sample-weighted random forests (SSWRF) to deal with class imbalance. An SVM classifier was trained and applied to estimate the weights of training samples. Then, the training samples with estimated weights were utilized to train sample-weighted random forests(SWRF). They extracted three types of fea-tures, PSSM, averaged cumulative hydropathy (ACH), and predicted RSA. The proposed SSWRF achieved 67.9% accuracy. Similarly, in the same year, the authors in Jia et al. (2016) proposed a Sequence-Based Ensemble Clas-sifier for Identifying PPIs by optimizing an imbalanced training dataset called iPPBS-Opt. They used the K-Nearest Neighbors Cleaning (KNNC) and Inserting Hypothetical Training Samples (IHTS) treatments to optimize the training dataset. They used the ensemble voting approach to select the most relevant features and the stationary wavelet transform to formulate the statistical samples. Two benchmark datasets were used for this study. One is the “surface-residue” dataset, and the other is “all-residue”. DSSP program (Hooft et al., 2008) was used to find surface residues, while the PSAIA program (Mihel et al., 2008) was used to find the interfacial residues. To optimize the unbalanced training dataset they used K-Nearest Neighbors Cleaning (KNNC) treatment to remove some redundant negative samples. Random Forest and Ensemble Classifier were used to train the dataset. They supplied a web server for the predictor with step-by-step guide to maximize the convenience of most experimental scientists.

Many approaches have integrated multiple models to achieve better performance. Kang et al. (2023) integrated CNN, MLP-Mixer, and LSTM models to create a hybrid network for PPI prediction (HNPPISP). The HNPPISP model combined a two-stage multi-branch network with an MLP-Mixer network, where the two-stage multi- branch network extracted global features and the MLP-Mixer network captured the long dependency among local features. Similarly, the authors in Hu et al. (2023) introduced D-PPIsite, an advanced deep learning model achieving an 87% accuracy rate integrating multiple DNNs. The predictor is available freely for academic use. Finally Aybey and Gümüş (2023), proposed SENSDeep, an ensemble learning framework that integrates the models of RNN, CNN, GRU sequence to sequence (GRUs2s), GRU sequence to sequence with an attention layer (GRUs2satt), and a multilayer perceptron. They added two more feature groups, which are secondary structure and protein sequence information, besides the current twelve groups. They proved that adding new features to the training data sets at the expense of data loss improves the prediction performance of the method and gives a similar performance with less data. In addition, considering the execution times, SENSDeep and its submodels seemed acceptable, although the trainings were carried out using processors only. It has been observed that these times have decreased considerably in the voluntary trials with GPUs.

Recently, data structures such as graphs have been recognized as one of the most convenient and intuitive ways to represent residues in a protein and their interactions. Alkhateeb and Awad (2024) trained a GCN model on protein interactions modeled as structured graph data, which allowed capturing dependencies between neighboring proteins more effectively than traditional models. Their approach extended the feature space with specialized input, yielding promising results. In the same direction, the authors in (Feng et al., 2024) introduced DGCPPISP, a two-stage transfer learning framework based on dynamic GCN. The main contributions of this study included the encoding of the target sequence in the first stage of transfer learning using the ESM-2(a protein pre-trained language model (PLM)) (Lin et al., 2022), coupled with four other sequence features as input to the training model. They used a protein-peptide binding residue dataset that is helpful for PPI prediction. By leveraging dynamic graph convolution modules, they addressed limitations in traditional GNN-based approaches.

**FIGURE 12:**
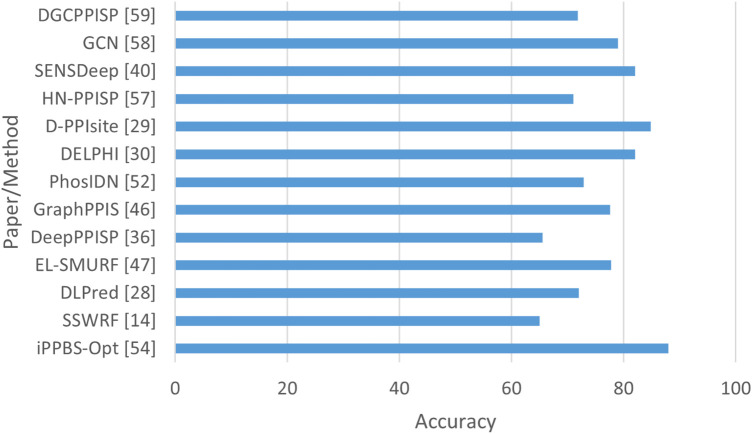
Performance analysis of highest accuracy reported by various papers of Approach A (in %).

In addition, recent advances showed a shift from isolated architectures (CNN, RNN, GCN) toward hybrid and multimodal PPI frameworks. Models such as SENSDeep (Aybey and Gümüş, 2023) integrated CNN, RNN, and attention mechanisms to capture both local and contextual dependencies. Moreover, the advent of PLMS such as ProtBERT, ESM-1b, and ESM-2 has transformed PPI prediction by enabling transfer learning from large-scale protein corporation. EGRET (Mahbub and Bayzid, 2022) represented an important shift toward hybrid and multimodal deep learning approaches for PPI prediction. Unlike early sequence-based CNN and RNN models, EGRET utilized a graph representation of proteins, where residues are modeled as nodes connected based on structural or spatial proximity. Using edge-weighted graph attention networks (GATs), the model was able to learn how to prioritize biologically meaningful residue relationships. EGRET combined evolutionary features with graph topological features, demonstrating that integrating sequence + structure information improved generalization performance in PPI site prediction. EGRet also followed the recent progression toward representation learning PLMs which generated rich residue-level embeddings from protein sequences by fusing PLM-derived sequence embeddings with graph-based structural encodings. Thus, EGRET can be considered a bridge model between classical handcrafted feature approaches and modern transformer-based multimodal frameworks in structural bioinformatics. These models generated contextual embeddings that can be integrated with CNN or GCN backbones to capture both sequence semantics and topological features, for example, DGCPPISP (Feng et al., 2024) leveraged ESM-2, a transformer-based PLM, within a dynamic GCN framework for improved generalization. In addtion HN-PPISP (Kang et al., 2023) employed graph attention and MLP-Mixer hybrids for 3D structure-based and sequence-based PPIs. Therefore, while CNNs, RNNs, and GCNs remain essential, their integration with PLM-derived representations marks a significant advance toward more generalizable and interpretable predictive models. Figure 12 presents the best performance in terms of accuracy with the most suitable parameter settings of the various deep learning approaches to predict PPIs in isolated protein sequences and using different benchmark datasets. We can observe that D-PPIsite (Hu et al., 2023), iPPBS-Opt (Jia et al., 2016), and SENSDeep (Aybey and Gümüş, 2023) achieved the best prediction accuracy in DNN, and EL, respectively. For more details, see Table 4.

**TABLE 4:** Performance measures for PPIs in Approach A.

Deep learning method	Name	Datasets	SPE	SEN	PRE	ACC	F-measure	MCC	AUC
DNN	DLPred (Zhang B et al., 2019a)	C	0.76	0.491	0.312	71.1	38.1	21.4	78.9
		B A	0.779 0.747	0.553 0.556	0.401 0.285	73.1 71.8	46.5 37.7	29.8 23.8	81.1 80.1
		D	0.787	0.554	0.418	73.68	47.61	31.4	81.81
	D-PPIsite (Hu et al., 2023)	C	0.86	0.39	0.39	77.8	38.6	25	71
		B	0.92	0.30	0.30	85.1	29.9	21.6	74
		A	0.89	0.37	0.37	80.9	37.3	26	73.2
		E	0.92	0.48	0.48	85.9	48	39.9	82.4
		F	0.93	0.46	0.46	87.1	46	38.7	82.2
	PhosIDN (Yang et al., 2021)	G	-	0.508	0.909	72.9	65.2	51.0	94.0
CNN	DeepPPISP (Zeng et al., 2020)	C							
		B	–	0.577	0.303	65.5	39.7	20.1	–
		A							
	HN-PPISP (Kang et al., 2023)	A, B, C	-	0.632	0.324	66.7	42.7	24.4	36
		K		0.449	0.253	76.2	32.4	20.4	25.3
EL	ELSMURF (Wang X et al., 2019)	C	0.76	0.8	0.77	77.7	78.2	55.4	88.7
		B	0.73	0.79	0.75	77.1	77.5	54.2	85.4
		A	0.78	0.81	0.79	79.1	78.4	58.4	88.5
	SSWRF (Wei et al., 2016)	C	0.65	0.53	0.32	62.1	36.5	15.2	
		B	0.64	0.65	0.27	64.8	35.1	22.4	71.1
		A	0.70	0.58	0.32	67.9	38.6	23.4	
	iPPBS-Opt (Jia et al., 2016)	surface-residue	0.94	0.58	-	89.34	-	58.21	89.34
		all-residue	0.97	0.39		88.20		46.62	88.20
	SENSDeep (Aybey and Gümüş, 2023)	A	0.858	0.431	0.357	79.3	38.9	26.8	72.6
		B	0.832	0.448	0.258	78.8	32.7	22.4	71.4
		C	0.866	0.355	0.363	77.6	35.8	22.3	68.5
		E	0.894	0.34	0.342	81.9	33.8	23.5	68.0
		F	0.898	0.361	0.329	83.4	34.1	24.9	69.2
	DELPHI (Li et al., 2021)	A	0.884	0.351	0.351	80.3	35.1	23.5	71.0
		C	0.857	0.352	0.352	76.5	35.2	20.9	68.5
		B	0.914	0.274	0.274	84.7	27.4	18.9	71.1
		E	0.901	0.371	0.371	82.9	37.1	27.2	73.7
		F	0.914	0.364	0.364	84.8	36.4	27.8	74.6
GCN	GraphPPIS (Yuan et al., 2021)	Test_60	-	0.584	0.368	77.6	45.1	33.3	78.6
		C							
	EGRET (Mahbub and Bayzid, 2022)	B	0.715	0.561	0.358	71.5	43.8	27	71.9
		A							
	DGCPPISP (Feng et al., 2024)	A, B, C, E, K	-	0.617	0.372	71.8	46.4	30.6	44.6
	GCN (Alkhateeb and Awad, 2024)	A, B, C	-	0.45	0.74	79	49.0	-	-

### 4.2 Approach B: PPI prediction of pair of proteins

Unlike the approaches that infer interactions from isolated protein sequences, studying PPIs in pairs allows a direct examination of binding events and interaction dynamics. In addition, it provides detailed insights into the specificity and regulation of these interactions. This section reviews state-of-the-art computational models that integrate protein sequences, structural, and network information to predict and validate protein interactions. The use of DL algorithms in PPIs prediction tasks began in 2017 when Sun et al. (2017) proposed the use of a stacked autoencoder (SAE) to filter heterogeneous features in a low-dimensional space. The protein sequences were numerically represented using auto-covariance (AC) and conjoint triad (CT) methods. The representation of each protein was then fed to a DNN model for training with ten-fold cross-validation. The authors observed that with a one-hidden-layer, both DNN models attained high accuracy. The authors concluded that the accuracy of a model does not require a complicated network with a large number of layers and neurons. In the final model construction, they trained the DNN model on the entire benchmark dataset using AC features, which had better accuracy. Finally, they compared their results with other ML approaches that used the same dataset and showed the superiority of their method. Very next in the same year and following a similar pattern, Du et al. (2017) employed the five widely used descriptors, namely AAC, DPC, QSO, APAAC, and composition/transition/distribution, to represent the protein sequence, which is then effectively learned by a DNN model named DeepPPI. The authors presented the performance of DeepPPI using two different network architectures: one by connecting the two inputs in a single network; and another using two networks for each protein separately. Finally, they evaluated their model using a 5-fold CV after setting the network with the best hyperparameters. DeepPPI seemed superior in terms of accuracy and running time on all other existing approaches: SVM, AdaBoost, and RF.

The authors in Li et al. (2018) presented DNN-PPI: a generalization tool for PPI prediction for the first time. They used Pan’s human PPI dataset for training. They built several validation datasets from four well-known PPI data sources for validation. They evaluated the performance of the model using datasets from external species. The different types of features, including semantic associations between amino acids, position-related sequence segments (motif), and their long- and short-term dependencies, were captured in the embedding, CNN, and LSTM layers, respectively. The prediction results obtained by DNN-PPI proved that it is a remarkable generalization tool for identifying protein interactions. Furthermore, with the intention of the generalization, a remarkable DL approach (DPPI) was implemented by Hashemifar et al. (2018) to handle large training data effectively and capture the potential features of protein pairs. The successful execution of the three main modules contributes to the design of the DPPI model. The first and core module is the convolutional module, which consists of a set of filters (convolutional layer, ReLU, batch normalization, and pooling layer) responsible for mapping the protein sequences to a representation suitable for further processing by detecting patterns that characterize the interaction information. The input in DPPI was taken as the sequence profiles, which were generated based on probability using the PSI-BLAST algorithm. The next module is Random Projection (RP), which consists of two FC sub-networks and is responsible for projecting the convoluted representation of two proteins to two different spaces. The word ‘random’ is used to take the random weights so that the model can learn motifs with different patterns. The outcome of the RP module is the refined representation of the proteins, which is then taken as the input by the last module, i.e., the prediction module. The prediction module computes the probability score by performing the element-wise multiplication on the representation taken from the previous module, which indicates the interaction probability of two proteins in a pair. This Siamese-like CNN behaved very well when evaluated with different benchmark datasets. The authors committed that DPPI can serve as a principal model for sequence-based PPI prediction and is generalizable to diverse applications.

Inspiring the advances of ML approaches, the authors in Wang et al. (2017) predicted the interactions among proteins by combining the ensemble RF classifier and the discrete cosine transform (DCT) algorithm. They calculated the PSSM matrix from the alignment of amino acid sequences, and then the feature vector was computed using DCT to present protein evolutionary information. Their method achieved excellent results. They applied their model to independent data sets and achieved good prediction accuracy. Compared with the SVM method, this model had better performance. In addition, in the same trend, Wang L. et al. (2019) leveraged CNN to deeply extract hidden features from matrix-based biological information of the protein generated by the PSSM matrix. Then, the prediction task was accomplished by proposing a Feature-Selective Rotation Forest algorithm (FSRF), whose main purpose is to reduce data dimension and noisy information, and to improve the prediction accuracy and the running time. The proposed approach was experimented on two realistic datasets, namely Yeast and *Helicobacter Pylori*. To further evaluate the prediction performance, they compared the results of CNN-FSRF with SVM and other methods. In addition, they tested CNN-FSRF on other independent datasets and achieved favorable results. The authors in Zhang et al. (2023) combined two-dimensional CNN models to develop DeepSG2PPI. They calculated the protein sequence and the local context information of each amino acid residue. Then, they extracted features from a two-channel coding structure using a two-dimensional CNN (2D-CNN) model. In the 2D-CNN model, an attention mechanism is embedded to set higher weights to key features. The final biological features of the protein are represented as a graph embedding vector, which includes the global statistical information of each amino acid residue and the relationship graph between the protein and Gene Ontology (GO). Finally, a 2D-CNN model and two 1D-CNN models are combined for PPI prediction. Comparison analysis with existing algorithms showed that the DeepSG2PPI method has outstanding performance, providing more accurate and effective prediction of PPI, which can help reduce the cost and failure rate of biological experiments. Similarly, using multiple DNNs, Zhang L. et al. (2019) introduced EnsDNN, an ensemble DNN-based approach for PPI prediction. In EnsDNN, three different feature sets are generated based on auto-covariance (AC), local descriptor (LD), and multi-scale continuous and discontinuous local descriptor (MCD). For each set of features, they trained nine independent DNNs with different configurations and parameter settings. The final 27 trained DNNs were ensembled to form a two-layer NN for the prediction. This strong and capable ensemble predictor leveraged the advantages of key information about interaction generated by the three different feature extraction approaches and an assortment of 27 DNNs. The model attained remarkable performance when evaluated on training datasets as well as independent datasets.

Employing the features of RNNs, Richoux et al. (2019) proposed a fully connected model and a recurrent model to compare two different neural network architectures. The dataset is extracted from the UniProt website. With regard to performance, the fully connected model achieved 76% accuracy and the recurrent model achieved 78% accuracy. The authors claimed that they conducted training and testing in strict conditions to build strong confidence in the ability of a model to scale to larger datasets. In another similar approach, Chen et al. (2019) attempted to capture the mutual influence of the protein pairs in PPI prediction based on a Siamese architecture (PIPR). Besides the binary prediction, PIPR addressed the issues of the estimation of binding affinity and the prediction of interaction type. PIPR incorporates a deep Siamese environment of a residual RCNN-based protein sequence encoder to better apprehend the potential features for PPI representation. This deep encoder comprises many occurrences of convolution layers with pooling and bidirectional residual gated recurrent units to ease the training and greatly diminish the updates of the parameters. For the numerical representation of the protein sequences, PIPR transformed the recognized amino acids based on their similarity in terms of co-occurrences as well as electrostatic and hydrophobic properties, and the pre-trained amino acid embedding. The resultant embeddings were then fed to the RCNN encoder to capture the latent information of the proteins. The output of the RCNN encoder, which is a refined embedding of the protein sequences, is then merged to generate a pair vector and passed into a multilayer perceptron (MLP) with Leaky ReLU for PPI classification. PIPR proved promising results by effectively covering the mutual influence among the protein pairs and ascertaining the generalization without the inclusion of hand-crafted features.

Following the same trend, the authors of Czibula et al. (2021) used a Siamese structure and proposed a binary supervised classifier (AutoPPI) to predict PPI. They built and trained two autoencoders (AE) for each class in the input data, namely, positive interaction and negative interaction. The feature vectors combined AC, CT, and PseAAC encodings. For each autoencoder, three NN architectures were developed: 1) Joint-Joint architecture, which takes the features of a pair of proteins as input and correspondingly returns the renovated features at the output; 2) Siamese-Joint architecture, which uses a shared encoder to compress the two proteins to learn latent space representation, which is finally combined and used to regenerate the pair; 3) Siamese–Siamese architecture in which a common representation is generated by element-wise multiplication of two encodings for each protein in a pair at the encoder side and the reconstruction of proteins is obtained using a shared decoder. In all three architectures, the SELU activation function and the Adam optimizer were used.

Considering the context features of protein sequence, the authors in Wang Y. et al. (2019) proposed a pure biological language processing model for predicting PPIs. Their CNN model was constructed based on a feature representation method for biological sequences called bio-to-vector (Bio2Vec. They used the Skip-Gram model (Mikolov et al., 2013b) to represent protein words. The prediction accuracy of their framework was 99.5%, which out-performed the latest methods. Such impressive results inspired other researchers to consider the context information and implicit semantic information of the bio-sequence. Following a similar pattern, the authors in (Jia et al., 2019) proposed a new predictor, called “iPPI- PseAAC(CGR)”, by incorporating the information of chaos game representation (CGR) into the PseAAC. They extracted the PseAAC and used the CGR to define the pseudo components. Finally, they applied the random forest and ensemble classifier to perform the prediction. They achieved around 92.95% accuracy in the benchmark datasets. A user-friendly web server has been published with this predictor. Further in ensemble methods (Chen et al., 2020), proposed an ensemble model called StackPPI to predict PPIs. They used XGBoost to eliminate the noise and reduce the dimensional-ity, which enhanced StackPPI’s performance. Finally, they built a stacked ensemble classifier that employs Random Forest and extremely randomized trees (ET) as the base-classifiers, and logistic regression (LR) as the meta-classifier. The distinct feature of this model is its ability to infer biologically significant PPI networks. StackPPI’s accurate prediction of functional pathways made it the logical choice for studying the underlying mechanism of PPIs, especially in drug design. Starting from 2020, the researchers involved the graphs in the PPI problems of pairs of proteins. The authors in Yang et al. (2020) involved Structural information of PPI networks, such as their degree, position, and neighboring nodes in a grap,h with the sequence information to be informative in PPI prediction. Facing the challenge of representing graph information, they introduced an improved graph representation learning method. Their model can study PPI prediction based on sequence information and graph structure. Moreover, their approach takes advantage of a representation learning model and employs a graph-based deep learning method for PPI prediction, which showed superiority over existing sequence-based methods. Followed by that, in 2022, the authors in Baranwal et al. (2022) developed a mutual graph attention network and a corresponding computational tool, Struct2Graph, to predict PPIs solely from 3D structural information. Struct2Graph used a graph-based representation of a protein globule obtained using only the 3D positions of atoms. This graph-based interpretation allows for neural message passing for efficient representation learning of proteins. A GCN maps graphs to real-valued embedding vectors in such a way that the geometry of the embedding vectors reflects similarities between the graphs. They achieved around 99% accuracy. This model can identify residues that likely contribute to the formation of the protein–protein complex. The identification of important residues is tested for two different interaction types: (a) Proteins with multiple ligands competing for the same binding area, (b) Dynamic protein-protein adhesion interac-tion. For applying DNNs on Human Protein, the authors in Le and Kha (2022) proposed a novel method to realize PPI prediction utilizing the FASTA (Pearson format) of amino acids. Compared with other ML methods, their DNN model achieved higher prediction accuracy using five-fold cross-validation. By evolving self-attention models, the authors in Li et al. (2022) proposed SDNN-PPI, a PPI prediction method based on self-attention and deep learning. The method adopts AAC, CT, and AC to extract global and local features of protein sequences, and leverages self-attention to enhance DNN feature extraction to more effectively accomplish the prediction of PPIs. Satisfactory results were obtained on interspecific and intraspecific datasets, and good performance was achieved in cross-species prediction. Recently, in 2023, the authors in Tran et al. (2023) proposed a DeepCF model that combines the learned features and handcrafted features for the first time. They utilized 5 protein sequence extractors: AAC, PseAAC, APAAC, QSO, and DPC, to extract handcrafted features, then applied a natural language processing technique, Word2vec, to generate learned features by embedding protein sequences into the feature space. Finally, a DNN architecture was employed for combining two types of features and identifying PPIs. DeepCF was evaluated on the Yeast core, Human, and eight independent datasets. The experimental results demonstrated the superiority of DeepCF over other methods.

Recent research has increasingly focused on hybrid and multimodal frameworks that integrate complementary neural components. For instance, CNN + GCN hybrids leverage convolutional layers to extract local residue features while graph convolutions capture global structural dependencies, improving spatial awareness in PPI prediction. Similarly, RNNs enhanced with attention mechanisms or transformer-style encoders have demonstrated strong capability to model long-range residue dependencies and contextual relationships. Such combinations outperform traditional sequence-based encoders and highlight a shift toward transformer-based multimodal approaches in current PPI research. For instance, SDNN-PPI (Li et al., 2022) employed self-attention to refine DNN feature extraction, and DeepCF-PPI (Tran et al., 2023) combined handcrafted descriptors with learned sequence embeddings (Word2Vec). In addition, Struct2Graph (Baranwal et al., 2022) explored graph attention and MLP-Mixer hybrids for 3D structure-based and sequence-based PPIs.

**FIGURE 13:**
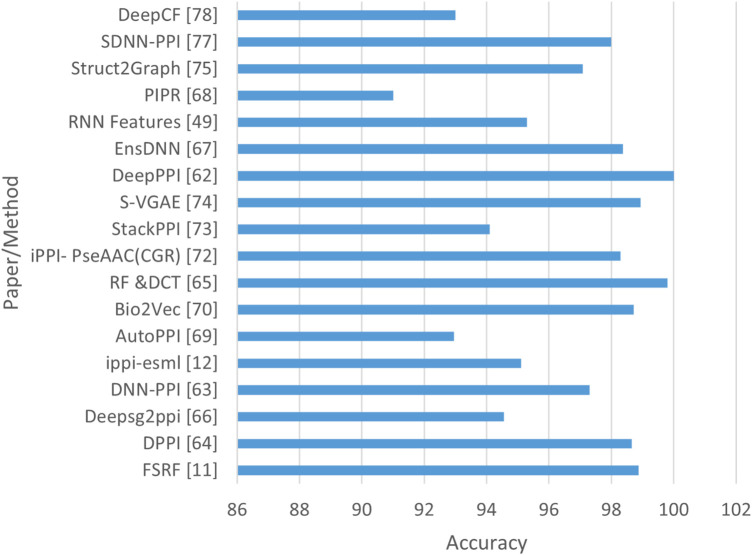
Performance analysis of the highest accuracy reported by various papers of Approach B (in %).

**FIGURE 14:**
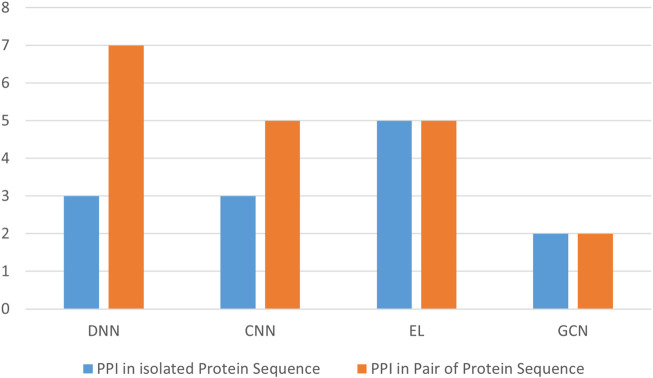
The number of papers published using a particular approach.

**FIGURE 15:**
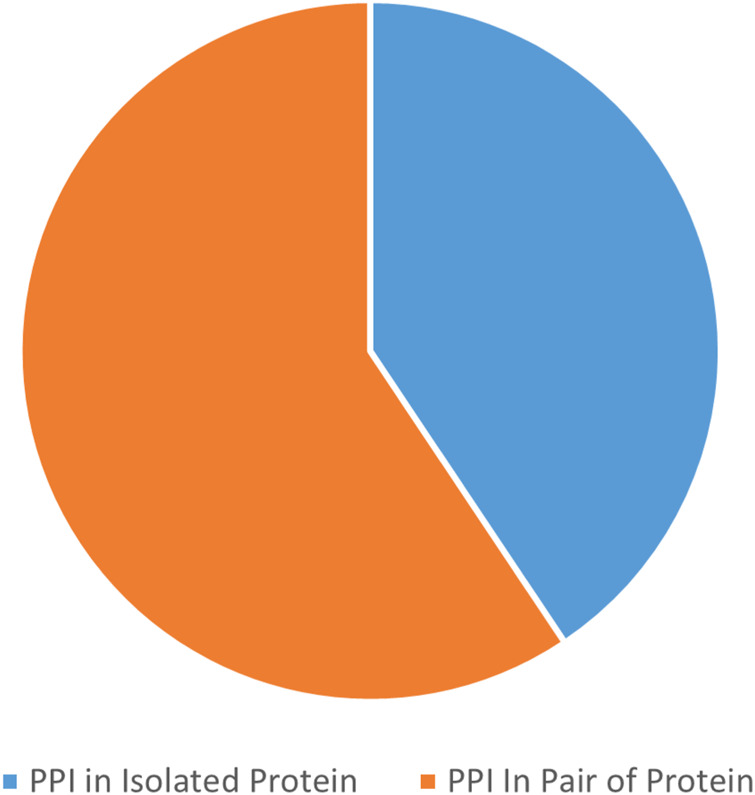
Number of published papers by DL in PPIs prediction.

Figure 13 presents the best performance in terms of accuracy with the most suitable parameter settings of the various deep-learning approaches to predict PPIs in pair of protein sequences. It can be observed that the prediction accuracy is high (*≥*90%), and DeepPPI has achieved the highest accuracy on benchmark datasets. Figure 14 illustrates the number of published research papers employing various DL models in PPI prediction. As shown, most studies utilized DNNs and EL, with a smaller number adopting CNNs, and only a few incorporating graph networks. Despite their limited representation, graph networks have demonstrated promising results, making them a highly promising venue for future research in the field of PPI prediction. Figure 15 presents the number of research papers that were published using a particular approach. We can observe that deep learning (DL) techniques were successfully used for both approaches; however, they were more popular in the prediction of pairs of proteins datasets (Approach B).

### 4.3 Experimental reproducibility

#### 4.3.1 Implementation environment

Most PPI deep learning frameworks utilized either PyTorch or TensorFlow, with hardware setups that include NVIDIA GPUs (Tesla V100, A100, or RTX 3090). The training epochs ranged from 50 to 300, depending on the dataset size and convergence behavior.

#### 4.3.2 Feature preprocessing

Feature extraction plays a central role in reproducibility:

- PSSM and Evolutionary Features: most of the methods, like SSWRF, DLPred, DeepPPISP, and CNN-FSRF, generated the PSSM using PSI-BLAST with default parameters of e-value = 0.001, BLOSUM62 substitution matrix, and 3 iterations against the NR (non-redundant) database.
- 3D-1D Features: derived using tools such as SPIDER3 (Heffernan et al., 2018), like in HN-PPISP or DSSP, like in DELPHI and DeepPPISP, encoding solvent accessibility and secondary structure probabilities into 1D descrip-tors.
- Residue Conservation and Evolutionary Conservation: most of the methods like DELPHI, D-PPIsite, HN-PPISP employed Consurf (Armon et al., 2001) or Rate4Site (Pupko et al., 2002) algorithms, aligning multiple homologous sequences to infer evolutionary conservation scores.
- Physicochemical Descriptors: Generated through ProPy (Cao et al., 2013) like in iPPBS-Opt and D-PPIsite or iFeature (Chen et al., 2018) like in EL-SMURF, including hydrophobicity, charge, and polarity scales.

#### 4.3.3 Hyperparameter settings

To ensure experimental reproducibility, we summarized and analyzed the hyperparameter configurations of the reviewed models in Table 1. Across most CNN-based and DNN-based architectures, the Adam optimizer was the choice, typically using learning rates around 0.001 and dropout rates between 0.2 and 0.7 to reduce overfitting. Models such as DeepPPISP and DELPHI used moderate batch sizes (32–64) and cross-entropy losses, while hybrid models like DLPred and PhosIDN employed multiple hidden layers and dropout regularization for better stability on small datasets. In graph-based frameworks like DeepGCN and DGCPPISP, learning rates were reduced further (0.0001–0.01) with 3–5 hidden layers, ReLU or LeakyReLU activations, and batch normalization to stabilize convergence. Ensemble learning approaches, including EL-SMURF, EnsDNN, and StackPPI, integrated varied configurations of base classifiers or neural sub-networks trained under diverse dropout and feature window settings, providing robustness against imbalance and overfitting. Models leveraging transfer learning, like EGRET and DGCPPISP, combined pretrained embeddings such as ProtBERT or ESM-2 with task-specific fine-tuning, often requiring fewer epochs but larger feature dimensions. Overall, while most studies converged on standard hyperparameter ranges (learning rate 0.0001–0.01, dropout 0.2–0.7, batch size 32–256), explicit reporting remained inconsistent, underscoring the importance of standardized reproducibility guidelines for future PPI prediction research.

## 5 Comparative assessment

### 5.1 Datasets

#### 5.1.1 Approach A: PPIs in isolated protein sequence

Three widely benchmarked datasets are used in PPI prediction of isolated protein sequence: Dset 186, Dset 72 (Murakami and Mizuguchi, 2010) and Dset 164 (Singh et al., 2014). The distribution of the datasets is relatively unbalanced, with positive samples accounting for only 10%–18% of the total sample size, which poses a challenge for the generalization of the model. Although deep learning models can effectively deal with the overfitting problem caused by data imbalance, most of these computational methods are very unstable and poorly generalized for these highly unbalanced benchmark datasets, which implies some room for improvement. Table 2 summarizes the main datasets used in PPI prediction. Dset 186 is built from the protein data bank (PDB) and consists of 186 protein sequences extracted from 105 heterodimeric protein complexes with a sequence identity <25% and a resolution of *≤* 3.0Å. Dset 186 has a total of 36216 residues (including 5551 interacting residues). Dset 72 and PDBset 164 are constructed in a way similar to the construction of Dset 186. Dset 72 contains 72 protein sequences from 36 protein complexes in the protein-protein docking benchmark set version 3.0. While under construction, all sequences in Dset 72 that have *≥* 25% sequence identity over a 90% overlap with any of the sequences in Dset 186 are removed. It contains 17975 residues in total, with 3799 interacting residues. Dset 164 consists of 164 non-redundant protein sequences with the same filtering requirement as for Dset 186. There are 6111 interacting residues and a total of 33678 residues in Dset 164. These datasets are used for training and testing deep learning models. Zhang B. et al. (2019) applied the DLPred predictor to the independent heteromeric dataset Dset 48, which is a subset of Dset 72, and five homodimeric sequences, to evaluate the DLPred model as a more general predictor. The study in (Hu et al., 2023) added Dset 448 and Dset 335 datasets to evaluate the performance of their model (D-PPIsite). Dset 448, which includes 448 protein sequences, is collected from the BioLiP database (Yang et al., 2012). The sequence identity between any two sequences in Dset 448 is less than 25%. Dset 355 was generated in DELPHI (Li et al., 2021) via removing the 93 redundant proteins from Dset 448. Furthermore, they compiled a large dataset of 9982 non-redundant protein sequences, including 427,687 binding and 3,826,511 non-binding residues. The maximum sequence identity between any two protein sequences in this dataset is 25%. Finally, they randomly selected 841 protein sequences to constitute the validation dataset, and the remaining proteins were used in the training dataset. The authors in (Kang et al., 2023) combined the three benchmark datasets and constituted one fused dataset called Dset 186 72 PDB164. In addition, they reduced Dset 448 and produced the Dset 331 with 331 valid proteins in total. They divided the two datasets into a test set and a training set according to a ratio of 1:6, respectively. Jia et al. (2016) used imbalanced datasets for their approach on PPIs prediction; they did not use any of the benchmark datasets. Instead, they extracted two datasets: the surface-residue dataset and the all-residues dataset. The protein-protein interfaces are usually formed by those residues that are exposed to the solvent after the two counter parts are separated from each other. The work in (Yuan et al., 2021) integrated three datasets, Dset 186, Dset 72 and Dset 164, into a fused dataset and removed the redundant proteins with more than 25% sequence similarities over 90% overlap on either sequence as in Dset 186 and obtained 395 protein chains, from which they randomly selected 335 protein chains for training (Train 335) and used the remaining 60 chains as independent test (Test 60). To further improve the stability and generalization performance of the models, an ensemble learning methods are applied to deal with the skewed distribution of categories in unbalanced datasets like (Wang Y. et al., 2019; Jia et al., 2016; Wei et al., 2016). DLPred is also a generalizable model and one of the most popular solutions to improve the performance of imbalance classification by applying the SLSTM Network (Zhang B. et al., 2019). Although most benchmark datasets in PPI prediction in isolated protein sequences focused on the annotated datasets extracted from the PDB database, several deep learning models in this survey have already utilized broader or disease-relevant resources. For example, EGRET (Mahbub and Bayzid, 2022) integrated sequence and structure data, and it was trained on multiple benchmark sets, such as Dset 186, Dset 72, and PDB164. These datasets include proteins from *H. pylori* and *E. coli*, covering both prokaryotic and eukaryotic species. GraphPPIS (Yang et al., 2020) was evaluated on Dset 331, which was derived from non-redundant PDB structures with diverse species origin (bacterial and eukaryotic). These cross-species datasets provide a valuable foundation for assessing generalization ability across biological domains. Table 4 concludes the datasets and the performance of each of them on PPIs prediction for isolated protein sequences.

#### 5.1.2 Approach B: PPIs in pair of protein sequences

There have been several benchmark datasets used to evaluate deep learning models trained on pairs of protein sequences. The S.cerevisiae dataset (You et al., 2014) is a core subset of the Database of Interacting Proteins (DIP). The positive and negative datasets are combined into a total of 11188 protein pairs. Martin et al. (2005) used the *Helicobacter pylori* proteins to construct a validation dataset, which is composed of 2916 protein pairs (1458 interacting pairs and 1458 non-interacting pairs). The study in Huang et al. (2015) constructed the Human dataset from the Human Protein Reference Database (HPRD). The Human dataset has 8161 protein pairs (3899 interacting pairs and 4262 non-interacting pairs). The authors in Zhou et al. (2011) collected five datasets: *Caenorhabditis elegans* (4013 interacting pairs), *Escherichia coli* (6954 interacting pairs), *Homo sapiens* (1412 interacting pairs), *Mus musculus* (313 interacting pairs), and H.pylori dataset (1420 interacting pairs). Sun et al. (2017) and Li et al. (2018) generated additional testing datasets from the 20160430 version of the Database of Interacting Proteins (DIP, Human). After the removal of common protein pairs from the benchmark dataset, 2908 pairs were obtained. Sun et al. (2017) used the HIPPIE dataset, release v2.0. It contains human PPIs from 7 large databases. They categorized the data, based on the PPI score, into “high quality” data (*≥*0.73) and “low quality” data (<0.73). After the removal of pairs shared with the benchmark dataset, they obtained 30074 high-quality interacting protein pairs and 220442 low-quality interacting pairs. The newly released InWeb inBioMap contains the human PPIs from 8 large databases. They screened out the PPIs with a “confidence score” equal to 1 as the “high quality” (HQ) data and treated the rest as the “low quality” (LQ) data. After the removal of pairs shared with the benchmark dataset, they identified 155465 of ‘high quality’ PPIs dataset and 459231 of “low quality” PPIs dataset. Martin et al. (2005) have generated the 2005-Martin dataset, which was used in other studies such as (Pan et al., 2010). (Richoux et al., 2019) retrieved human sequences from the UniProt database and split them into three datasets for training, validation, and testing. Li et al. (2018) added the *Drosophila* dataset, which contains 19133 positive samples and 18449 negative samples. Yeast dataset is used by Wang L. et al. (2019), Wang et al. (2017), and Chen et al. (2019). Baranwal et al. (2022) extracted a balanced dataset (consisting of an equal number of positive and negative pairs) and an unbalanced dataset (with a ratio of 1:10 between positive and negative pairs) from IntAct (Orchard et al., 2014) and STRING (Szklarczyk et al., 2019) databases. While most of these databases are compiled from eukaryotic model organisms such as *Saccharomyces cerevisiae* and *Homo sapiens* (human), emerging resources have broadened coverage to prokaryotes, virus–host systems, and disease-specific networks. For example, StackPPI (Chen et al., 2020) relied on datasets aggregated from IntAct and STRING, which have expanded their repositories to include archaeal and bacterial PPIs, such as those from *Escherichia coli* and *Mycobacterium tuberculosis*, which provide valuable information for studying essential metabolic pathways in prokaryotes. In addition, models such as SAE-based frameworks (Sun et al., 2017), DeepPPI (Du et al., 2017), and DNN-PPI (Li et al., 2018) relied heavily on the HIPPIE v2.0 and InWeb inBioMap datasets. HIPPIE computationally inferred PPIs from seven major databases (MINT, BioGRID, DIP, HPRD, IntAct, MIPS, and BIND) and categorizes them by reliability score. This scoring enables models to evaluate prediction stability across confidence levels and facilitates disease-specific network analysis. In particular, HIPPIE and InWeb annotate interactions with disease and tissue metadata, allowing researchers to map PPIs linked to cancer, cardiovascular, and neurodegenerative disorders. Several recent studies have exploited this property for model benchmarking and to explore context-specific sub-networks, such as Alzheimer’s disease-related interactomes (Ginsberg et al., 2022). virus–host interaction datasets such as VirHostNet 3.0 (Guirimand et al., 2015), IntAct Virus–Host (Brito and Pinney, 2017), and BioGRID COVID-19 (Oughtred et al., 2021) offer curated PPIs derived from experimental and text-mining sources, enabling the study of host–pathogen interface prediction via deep learning architectures. Although the models in this paper incorporate multiple datasets (e.g., Yeast, Human, *H. pylori*, *S. cerevisiae*, *E. coli*), we acknowledge that current benchmark collections still represent a limited biological spectrum. The diversity of protein structures, interaction mechanisms, and experimental biases remains a key constraint for evaluating deep learning models. Future studies should therefore focus on expanding dataset heterogeneity and establishing standardized cross-domain validation to ensure robust generalization. Table 4 presents the different datasets used for PPIs prediction of pairs of proteins and the performance of the deep learning model in each of them.

### 5.2 Performance measures

To quantify how correct the predictions made by an algorithm are, we used the following measures, including F1-score (F1), sensitivity (SEN), specificity (SPE), precision (PRE), accuracy (ACC), and Matthews correlation coefficient (MCC), see Equations 9–14.

$$F1-score=\frac{2\times TP}{2\times TP+FP+FN}$$ (9)

$$Recall=Sensitivity=\frac{TP}{TP+FN}$$ (10)

$$Specificity=\frac{TN}{TN+FP}$$ (11)

$$Precision=\frac{TP}{TP+FP}$$ (12)

$$Accuracy=\frac{TP+TN}{TP+TN+FP+FN}$$ (13)

$$MCC=\frac{\left(T|P|\times |T|n|-|F|P|\times |F|N\right)}{\sqrt{\left(TP+FP\right)\times \left(TP+FN\right)\times \left(TN+FP\right)\times \left(TN+FN\right)}}$$ (14)

where TP, TN, FP, and FN represent the numbers of true positive, true negative, false positive, and false negative residues in the prediction, respectively. Additionally, we reported the area under the receiver operating characteristic curve (AUC) to assess the overall predictive performance. Tables 4, 5 present the performance measures of the papers presented in Approach A and Approach B, respectively.

**TABLE 5:** Performance measurements for PPIs prediction in Approach B.

Deep Learning Method	Name	Datasets	SPE	SEN	PRE	ACC	F-measure	MCC	AUC
Deep neural networks	SAE (Sun et al. (2017))	C				99.21			
		D				97.14			
		E				93.77			
		F	-	-	-	92.24	-	-	-
		F				87.04			
		G				91.14			
		G				87.99			
	RNN (Richoux et al., 2019)	N	-	0.86	0.95	0.91	0.91	-	-
	DeepPPI (Du et al., 2017)	O		0.92	0.97	94.43		88.97	
		P		0.89	0.84	86.23		72.63	
		C		0.89	0.89	89.0	0.89		95.0
		Q		0.97	0.99	98.14		96.29	
	DNN-PPI (Li et al., 2018)	C				94.43		88.97	
		E				86.23			
		F				89.0			
		G				98.1			
		I		0.94	0.98	95.94	95.81	91.94	
		Q		0.97	0.99	98.38	98.37	96.81	
		K		0.98	0.99	98.66	98.64	97.32	
	DeepCF (Tran et al., 2023)	M		0.97	0.93	95		91	
		S		0.99	0.99	99		98	
		I		1	1	100		100	
	SDNN-PPI (Li et al., 2022)	O	0.97	0.93	0.97	95		91	98
		S	0.99	0.98	0.99	98		97	99
		Ab	0.96	0.96	0.90	93		86	98
		T	0.93	0.93	0.84	83		77	95
	DeepSG2PPI (Zhang et al., 2023)	N &STRING	0.98	0.98	0.98	0.98			
Convolution neural networks	CNN-FSRF (Wang L et al., 2019)	M	0.96	0.996	0.96	97.75	97.79		
		P	0.87	0.92	0.87	88.96	89.26		
		K		0.96		96.41	98.17	95.57	97.54
		I		0.95		95.47	97.68	78.09	87.08
		Q		0.99		98.65	98.32		
		R		0.93		93.27	96.52		
	DPPI (Hashemifar et al., 2018)	O		0.92	0.97	94.55			
	PIPR (Chen et al., 2019)	M	0.97	0.971	0.97	97.09	97.09	94.17	
	Bio2Vec-based (Wang Y et al., 2019)	C		0.96	0.98	97.31		94.76	99.61
		O		0.93	0.94	93.30		93.55	97.20
		P		0.88	0.88	88.01		87.9	93.94
		Ex-Human		0.996	0.995	99.58		99.16	99.95
Ensemble Learning	StackPPI (Chen et al., 2020)	P		0.88	0.90	89.27		78.59	
		O		0.93	0.96	94.64		89.34	
		Q				97.66			
		R				98.4			
		K				97.11			
		I				98.71			
	RF & DCT (Wang et al., 2017)	M		0.89	0.99	98.54		97.13	
		P		0.85	0.91	88.27		79.29	
		K				98.08			
		I				92.75			
		Q				98.87			
		R				98.72			
	iPPI-Esml (Jia et al., 2015)	P	0.88	0.90		90.75		81.51	
	EnsDNN (Zhang B et al., 2019)	O	0.95	0.95	0.95	95.29	95.29	90.59	97
		K				93.22			
		I				95.10			
		Q				95			
		P				89.14			
		R				94.06			
	iPPI-PseAAC(CGR) (Jia et al., 2019)	P	0.88	0.98		92.95		85.05	
		O	0.85	0.91		88.01		76.24	
	AutoPPI (Czibula et al., 2021)	C	0.99	0.92	0.97	97	97		97
		Multispecies	0.96	0.97	0.99	97	97		97
Graph convolutional network	Struct2Graph (Baranwal et al., 2022)	Balanced dataset(1:1)	0.994	0.986	0.994	98.96	98.98	97.91	99.62
		unbalanced dataset(1:2)	0.995	0.979	0.992	98.91	98.43	97.59	99.73
		unbalanced dataset(1:3)	0.996	0.974	0.988	99.01	98.12	97.46	99.7
		unbalanced dataset(1:5)	0.997	0.971	0.983	99.16	97.53	97.03	99.71
		unbalanced dataset(1:10)	0.997	0.956	0.970	99.26	96.31	95.90	99.54
	S-VGAE (Yang et al., 2020)	C,E,I,J,K				99.15	99.15		

### 5.3 Comparative performance of deep learning models for PPI prediction

**FIGURE 16:**
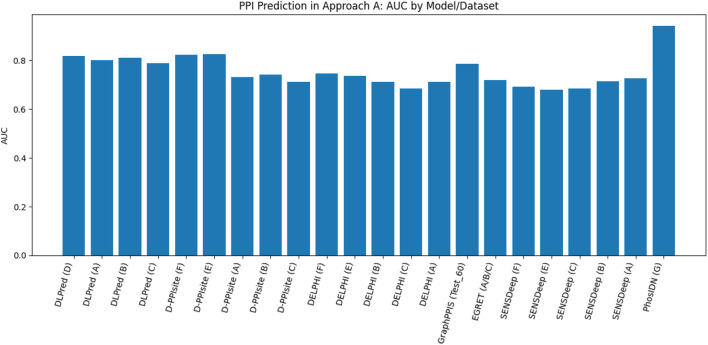
AUC values for the models in Approach A: AUC by Model/Dataset.

**FIGURE 17:**
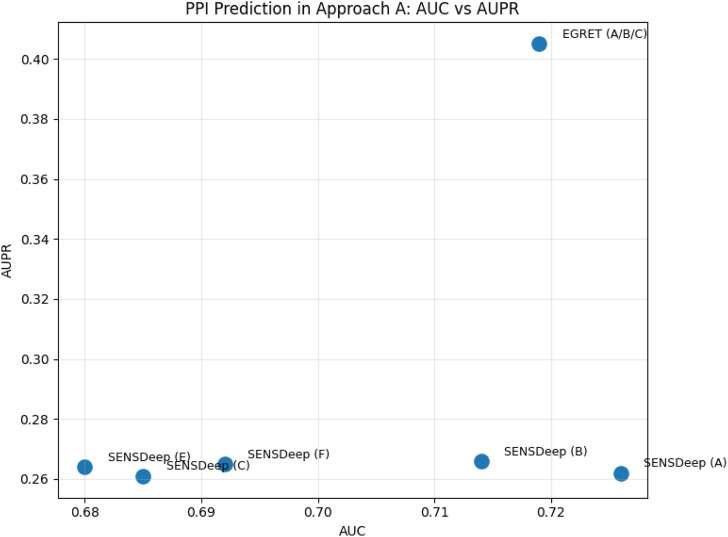
The comparative ROC and AUPR for the models in Approach A.

**FIGURE 18:**
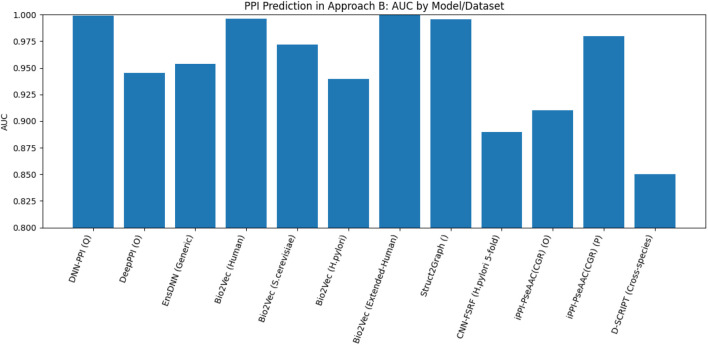
AUC values for the models in Approach B: AUC by Model/Dataset.

**FIGURE 19:**
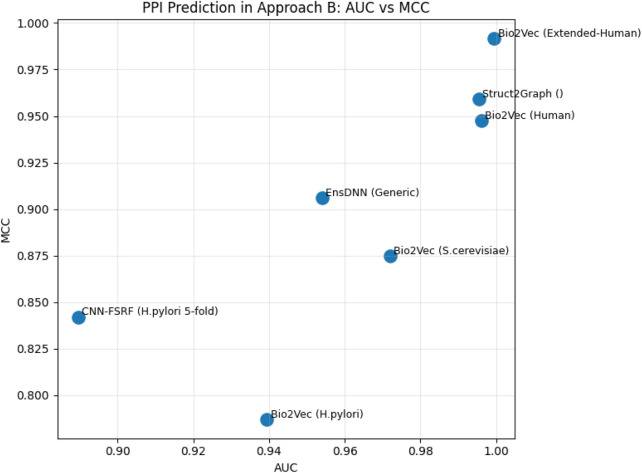
The comparative AUC and MCC for the models in Approach B.

Understanding the suitability of deep learning architectures for PPI prediction requires examining their inductive biases, data handling capabilities, and empirical stability across datasets. In PPI site prediction in isolated protein sequences, model performance strongly depends on the ability to capture sequential dependencies and spatial context. Traditional recurrent networks such as RNN and GRU effectively model short-term dependencies but exhibit vanishing gradient effects when capturing long-range residue correlations, resulting in limited recall (average sensitivity *≈* 0.30–0.45). Conversely, CNN-based architectures emphasize local motif learning through sliding windows, achieving moderate precision but often missing distal dependencies necessary for identifying the discontinuous binding residues. The SENSDeep ensemble addressed these limitations by integrating CNN, RNN, and attention-augmented GRUs (GRUs2satt) to com-bine both local and contextual information. On the Dset 72 dataset, SENSDeep achieved consistent gains across all folds with AUC *≈* 0.715 and AUPR *≈* 0.266, surpassing single encoders (AUC *≈* 0.69–0.71). This ensemble approach reduced prediction variance and enhanced robustness against class imbalance. When compared across the three annotated datasets (Dset 186, Dset 7, and Dset 164), Structure-aware CNNs (DELPHI and HN-PPISP) and hybrid GCN variants (EGRET and DGCPPISP) demonstrated progressive improvements in AUPR (0.36–0.45) and MCC (0.23–0.31), highlighting the contribution of spatial topology and pretrained embeddings (ProtBERT, ESM) in capturing non-local structural cues. For Pair-wise Protein Interaction Models (Approach B): Ensemble methods such as StackPPI and EnsDNN leveraged bagging and deep aggregation to mitigate imbalance, achieving AUC *≈* 0.96–0.97 and MCC *≈* 0.80–0.90. Deep-feature approaches like DeepPPI further integrated physicochemical descriptors and convolutional encoders, improving predictive stability with AUC *≈* 0.99 and MCC *≈* 0.97. Graph representations such as Struct2Graph transformed proteins into atomic-contact networks, achieving similar performance (AUC *≈* 0.995) while enhancing interpretability. Attention and feature-fusion frameworks extend this progress. CNNFSRF integrated CNN layers with feature-selection and random-forest fusion, and achieved AUC *≈* 0.89 on *H. pylori*. DeepCF-PPI, which combined learned embeddings with handcrafted features via attention, reported an AUC *≈* 0.97, an AUPR *≈* 0.978, andanMCC *≈* 0.90, confirming that hybrid attention mechanisms efficiently capture complementary biological information. Overall, attention-enhanced and graph-aware frameworks deliver superior generalization on unbalanced datasets by combining global reasoning with noise-tolerant feature fu-sion. The comparative ROC, AUPR, and MCC (Figures 16–19) visually confirm these trends for both isolated and pair-wise PPIs.

### 5.4 Transformer-based architectures and protein language models (PLMs)

Recent years have witnessed the rapid convergence of Transformer-based architectures with other deep architectures to enhance PPI prediction performance and interpretability. Transformer-based architectures such as ProtBERT (Gao et al., 2024), ProtT5 (Li et al., 2024), and the ESM (Evolutionary Scale Modeling) series (Xu, 2023) have been employed in protein representation and learning. These models are trained on billions of amino acid sequences and employ attention mechanisms to capture long-range dependencies and contextual relationships that are difficult to model with conventional DL architectures. Unlike convolutional sequence features, transformer-based embeddings encode deep contextual semantics that transfer effectively across diverse protein-related tasks, including PPI prediction, functional annotation, and structure modeling. For example, ProtBert-BiGRU-Attention (Gao et al., 2024) and P-PPI (Anteghini et al., 2023) frameworks demonstrated superior cross-species generalization compared to sequence-only methods such as DLPred and DeepPPISP, achieving AUC values above 0.90 on the yeast test set. Similarly, the EGRET model integrated ProtBERT-based embeddings with GAT layers, improving sensitivity and robustness in residue-level binding site detection. In addition, ProtBERT and ESM-2 were able to capture global contextual dependencies within protein sequences using self-attention mechanisms, pro-viding residue-level embeddings rich in biochemical and evolutionary information. These advances indicate a paradigm shift in PPI prediction, moving from task-specific architectures toward pretrained foundation models that can be fine-tuned for various interaction modalities. However, despite their remarkable repre-sentation power, PLMs remain computationally intensive, and they are often insufficient alone for modeling structural topology and intermolecular interactions. Therefore, hybrid models have emerged to integrate these embeddings with complementary technologies. Following this trend, Several recent frameworks employed PLM embeddings as node features in GNNs to learn sequence and structural relationships. For example, in Approach A: EGRET combined ProtBERT embeddings with graph attention networks to model residue-level spatial dependencies, while DGCPPISP integrated ESM-2 representations within a dynamic GCN to capture conformational flexibility. Similarly, in Approach B, GraphPPIS encoded structural proximity through weighted graphs enriched with PLM features. Such fusions significantly improved generalization in disease-specific PPI interaction predictions. In addition, the frontier of PPI research lies in multimodal architectures that unify diverse biological data based on sequence, structure, and multi-omics. Frameworks such as ProtST (Xu et al., 2023) and BioT5+ (Pei et al., 2024) embedded PLM-derived sequence features, AlphaFold (Faisal et al., 2025) predicted structural graphs, and co-expression signals from transcriptomic or proteomic data. By aligning modalities within a shared latent space, these models enhance biological interpretability and enable cross-species transfer learning. Authors in (Chinami, 2025) employed AlphaFold3-guided structural profiling of PPIs, integrating evolutionary distances and structural affinity metrics derived from predicted PPI complexes. They used PPI pairs from a cancer-wide interactome database with relevance to liver cancers. Their findings highlighted the power of integrative structural PPI mapping to uncover functionally significant distinctions in tumor biology and suggest a paradigm shift in cancer diagnostics enabled by next-generation structure-based analytics. Integrating PLMs with graph reasoning and omics data represents a promising route toward systems-level PPI inference and disease-specific interaction predictions. Collectively, these developments mark a paradigm shift from single-modality encoders toward context-aware, multimodal approaches, establishing a foundation for scalable and biologically grounded PPI discovery.

## 6 Limitations and future directions

Despite the remarkable progress in deep learning models for PPI prediction, current methods still have several limitations that restrict their generalization, interpretability, and biological transferability. In this section, we will discuss these limitations, focusing on the recent advances in this domain. Traditional machine-learning methods, such as RF, SVM, and Gradient Boosting, rely on manually designed descriptors and handcrafted feature extraction methods from the annotated datasets. While these models, such as RF-PPI (Hou et al., 2017) and SSWRF, are interpretable and computationally efficient, they fail to capture the higher-order dependencies between distant residues or conformational dynamics within the isolated protein surface. Early deep-learning models, such as DLPred and DeepPPISP, employed CNN and RNN architectures to automate feature ex-traction. However, CNNs suffer from limited receptive fields and tend to emphasize local patterns, while RNNs face gradient-vanishing issues and difficulty in learning long-range dependencies in long amino-acid chains. Consequently, both architectures struggle to model cooperative binding regions and generalize across species with significant sequence variation. To overcome these deficiencies, graph-based learning emerged as a powerful framework for encoding structural and relational information. Methods such as GraphPPIS and EGRET exploit graph-convolutional and attention mechanisms to propagate information across spatially proximal residues, capturing non-local structural dependencies. Nevertheless, the predictive performance of graph models can deteriorate on sparse or noisy interaction networks, and they remain sensitive to incomplete contact maps and imbalanced datasets. Ensemble methods, including StackPPI, SSWRF, and iPPBS-Opt, have been proposed to enhance robustness by aggregating multiple learners with complementary strengths. These models mitigate overfitting and bias by exploiting bagging and boosting strategies, improving stability and generalization in unbalanced or cross-domain PPI prediction tasks. Table 1 concludes the reported limitations of some of the discussed DL models. Recent developments in Transformer architectures have significantly improved biological sequence modeling. Transformers leverage self-attention mechanisms to capture global relationships, enabling the modeling of long-range dependencies that CNNs and RNNs fail to preserve. PLMs such as ProtBERT and ESMs models are trained on millions of protein sequences, allowing them to learn high-level representations that generalize across species and functional classes. When integrated into downstream PPI frameworks (e.g., EGRET), PLM-derived embeddings substantially enhance transfer learning performance and improve the detection of disease-related or virus–host interactions. These advances underline the transition from purely feature-driven models toward context-aware, cross-species, and multimodal architectures, capable of integrating sequence, structural, and functional modalities within a unified learning framework. However, these models are computationally heavy, require large GPUs, and their interpretability and biological correlation are still limited. Future research should focus on (1) scaling PLMs with structural alignment and contact-map supervision, (2) designing interpretable graph–Transformer hybrids to improve explainability, and (3) expanding benchmarking datasets beyond human and yeast to encompass archaeal, viral, and disease-specific PPIs. Such efforts will accelerate progress toward biologically faithful, generalizable, and clinically relevant PPI prediction.

## 7 Conclusion

The prediction of protein-protein interaction (PPI) hot spots plays a critical role in understanding molecular interactions, aiding drug discovery, and advancing computational protein design. This paper provides a comprehensive review of PPI prediction using sequence information and focusing on four architectures of deep learning: DNNs, CNNs, GCNs, and RNNs. In addition, we considered deep learning variants techniques under ensemble methods. We broadly discussed the various approaches in terms of input data, objectives, research contribution, extracted features, and the structure of the deep learning architecture, along with their best-suited parameters. While deep learning models have significantly improved predictive accuracy, challenges such as data imbalance, model interpretability, selecting for a suitable architecture with favorable hyperparameters, and integrating diverse biological information remain unresolved and have room for investigation. In addition, the emergence of graph-based models and hybrid deep learning architectures presents a promising direction for future research. The continued advances in feature engineering, model optimization, and large-scale dataset availability will further enhance the reliability and applicability of deep learning in PPI hot spot prediction. The in-depth, detailed discussion presented herein carefully mines every possible information, can help researchers to further explore the success in this area. We believe that this literature survey will benefit scholars in the applications of deep learning in the prediction of PPIs in imminent research.

## Glossary

DL: Deep Learning
PPI: Protein-Protein Interaction
DNN: Deep Nueral Network
CNN: Convolutional Neural Network
EL: Deep Ensemble Learning
GCN: Graph Convolutional Network
PBR: Protein-Binding Residues
PSSM: Position-Specific Scoring Matrix
RF: Random Forest
SVM: Support Vector Machine
RNN: Recurrent Neural Network
GPU: Graphics processing unit
GRU: Gated Recurrent Units
GNN: Graph Neural Network
HSP: High-scoring Segment Pair
SLSTM: Simplified Long Short-Term Memory
SAE: Stacked Auto-Encoder
AC: Auto Covariance Method
AAC: Amino Acid Composition
PseAAC: Pseudo-Amino Acid Composition
APAAC: Amphiphilic PseAAC
QSO: Quasi-Sequence-Order
DPC: Dipeptide Composition
ET: Extremely randomized Trees
DCT: Discrete Cosine Transform
AC: Auto covariance descriptor
MCD: Multi-scale continuous and discontinuous local descriptor
LD: Local Descriptor
CGR: Chaos Game Representation
GAN: Graph Attention Network
CT: Conjoint Triad
CV: Cross Validation
ML: Machine Learning
ReLU: Rectified Linear Unit
AF: Activation Function
LF: Loss Function
